# Supplementary material for: Fibroblast growth factor 18 stimulates the proliferation of hepatic stellate cells, thereby inducing liver fibrosis
Source: Nat Commun. 2023 Oct 9;14:6304. doi: 10.1038/s41467-023-42058-z (PMC10562492; doi:10.1038/s41467-023-42058-z)
Supplement: Supplementary file 1 — Supplementary Information [file 41467_2023_42058_MOESM1_ESM.pdf]

Fibroblast growth factor 18 stimulates the proliferation of hepatic stellate cells, thereby inducing liver fibrosis

Yuichi Tsuchiya[1,2*], Takao Seki[1*], Kenta Kobayashi[1,3], Sachiko Komazawa-Sakon[1], Shigeyuki Shichino[4], Takashi Nishina[1], Kyoko Fukuhara[5], Kenichi Ikejima[5], Hidenari Nagai[6], Yoshinori Igarashi[6], Satoshi Ueha[4], Akira Oikawa[7], Shinya Tsurusaki[8,9], Soh Yamazaki[1], Chiharu Nishiyama[3], Tetuo Mikami[10], Hideo Yagita[11], Ko Okumura[12], Taketomo Kido[13], Atsushi Miyajima[13], Kouji Matsushima[4], Mai Imasaka[14], Kimi Araki[15,16], Toru Imamura[17,18], Masaki Ohmuraya[14], Minoru Tanaka[8,9], Hiroyasu Nakano[1**]

Supplementary Information

Supplementary Figures 1-15

Supplementary Data 1-4

Supplementary Tables 1-4

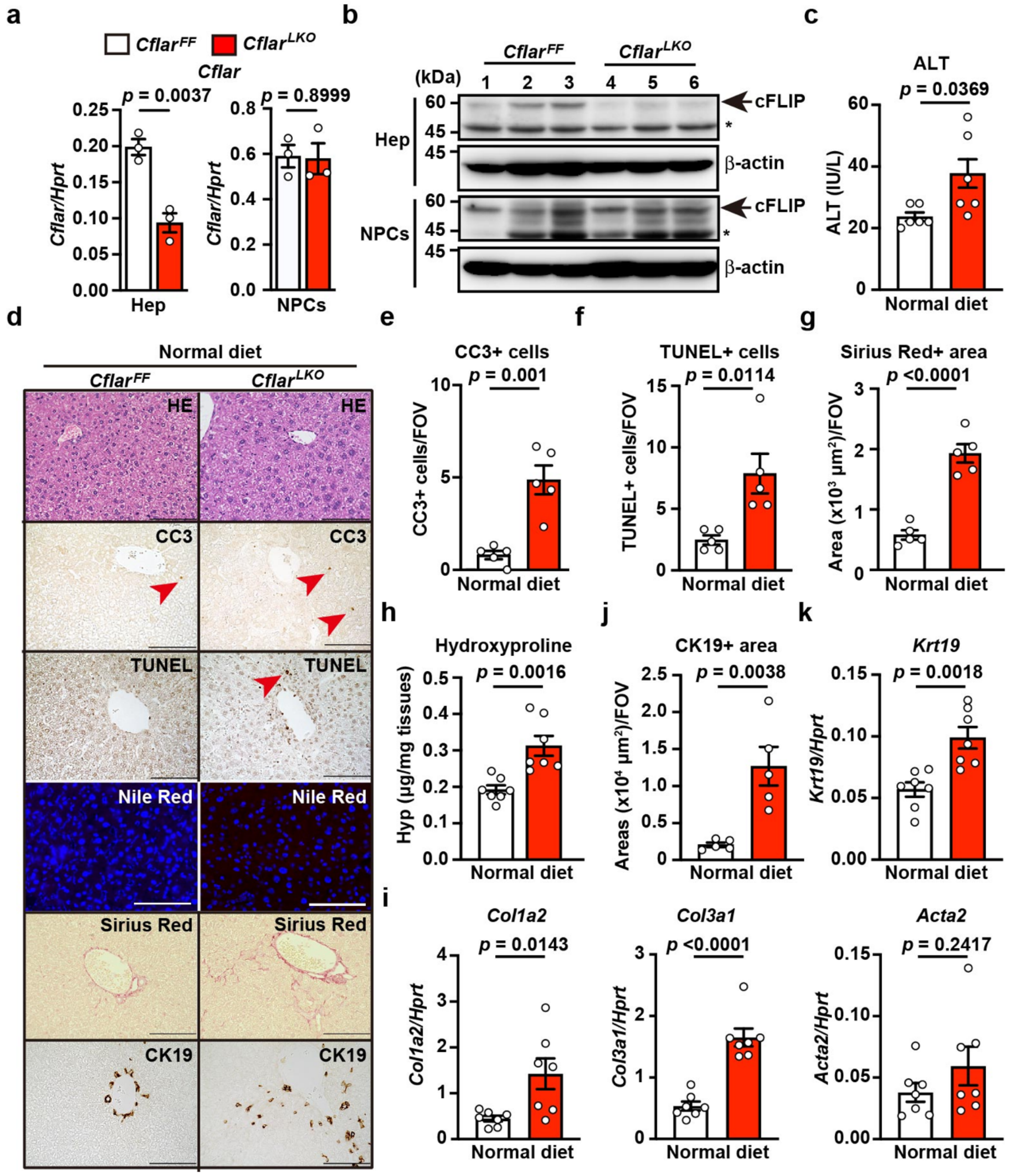

Supplementary Figure 1 | *Cflar*^{LKO} mice develop mild liver fibrosis. Eight-week-old *Cflar*^{FF} and *Cflar*^{LKO} mice were fed the normal diet. **a**, **b,** The expression of *Cflar* in hepatocytes, but not NPCs is decreased in *Cflar*^{LKO} mice compared to *Cflar*^{FF} mice. Hepatocytes and NPCs were prepared and the expression of *Cflar* was determined by qPCR (**a**). Results are mean ± SE (n = 3 mice). Expression of cFLIP in hepatocytes and NPCs was analyzed by Western blotting (**b**). Numbers indicate an individual mouse. Arrows and asterisks indicate cFLIP and cross-reacted bands, respectively. Results are representative two independent experiments (**a, b**). **c**, Serum ALT concentrations were

determined. Results are mean ± SE (n = 6 mice). **d-g, j**, Liver sections from mice of the indicated genotypes were stained with H&E, anti-CC3 antibody, TUNEL, Nile Red, Sirius Red, and anti-CK19 antibody (n = 5). Scale bar, 100 μm. Red arrowheads indicate $CC3^{+}$ or $TUNEL^{+}$ cells. The number of $CC3^{+}$ (**e**) and $TUNEL^{+}$ (**f**) cells were counted and expressed as in Fig. 1d. The Sirius Red^{+} (**g**), and $CK19^{+}$ (**j**) areas were quantified and expressed as in Fig. 1f. Results are mean ± SE (n = 5 mice). **h**, The hydroxyproline content of the livers was determined. Results are mean ± SE (n = 7 mice). **i, k**, The expression of the indicated genes in the liver was determined by qPCR. Results are mean ± SE (n = 7 mice). Pooled results of two or three independent experiments are shown (**c-k**). Significance was determined by the two-tailed unpaired Student's t-test. Source data are provided as a Source Data file.

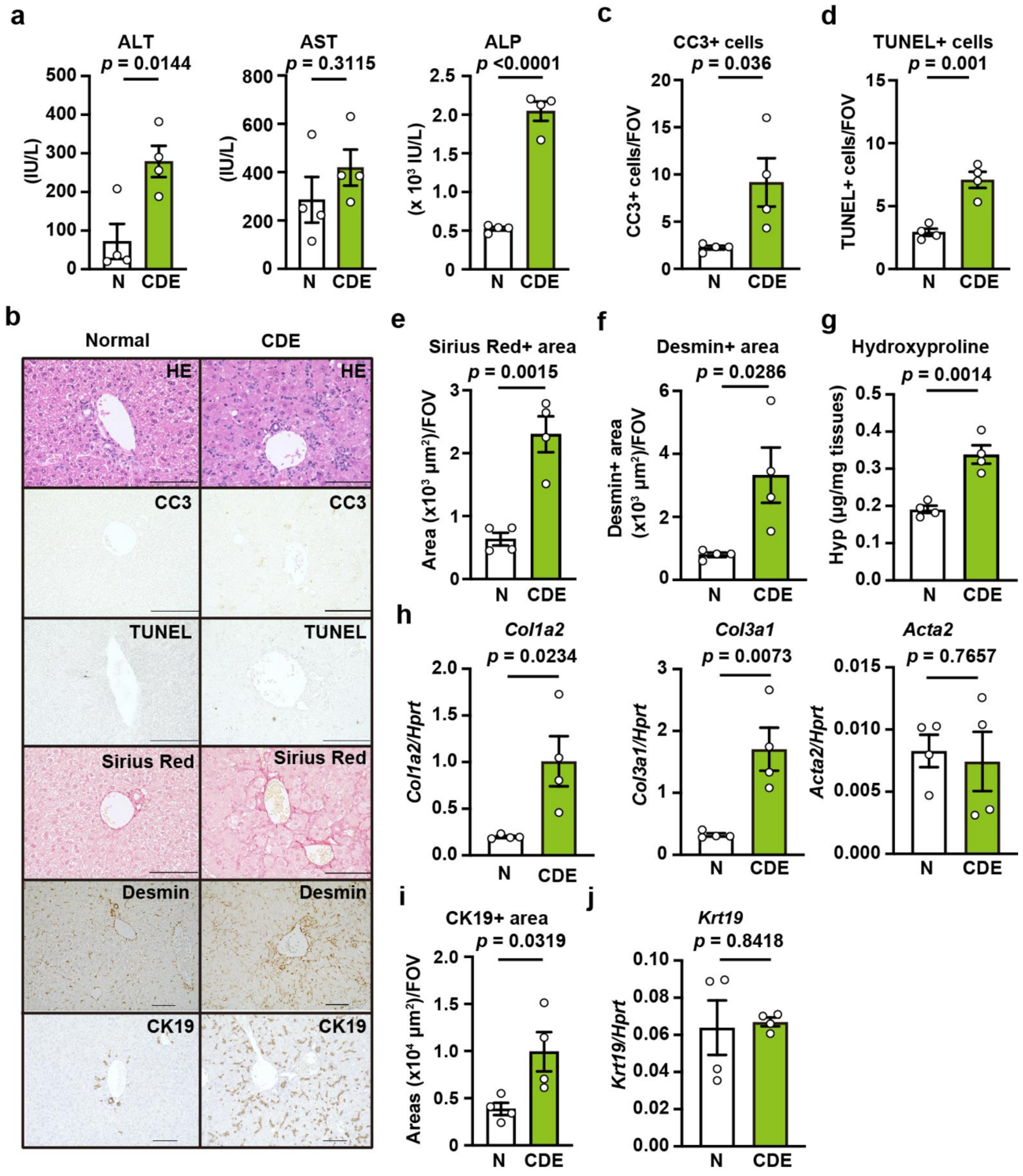

Supplementary Figure 2 | CDE diet induces liver fibrosis and ductular reaction in wild-type mice. Eight-week-old female wild-type mice were fed the normal diet (N) or CDE diet for 4 weeks. **a**, Serum ALT, AST, and ALP concentrations were determined. Results are mean ± SE ($n = 4$ mice). **b-e, f, i**, Liver sections were stained with H&E, anti-CC3 antibody, TUNEL, Nile Red, Sirius Red, anti-desmin antibody, and anti-CK19 antibody ($n = 4$). Scale bar, 100 μm. Red arrowheads indicate CC3$^+$ or TUNEL$^+$ cells. The number of CC3$^+$ (**c**) and TUNEL$^+$ (**d**) cells were counted and expressed as in Fig. 1d. The Sirius Red$^+$ (**e**), desmin$^+$ (**f**), and CK19$^+$ (**i**) areas were quantified and expressed

as in Fig. 1f. Results are mean ± SE (n = 4 mice). **g,** The hydroxyproline content of the livers was determined. Results are mean ± SE (n = 4 mice). **h, j**, The expression of the indicated genes in the livers was determined by qPCR. Results are mean ± SE (n = 4 mice). Results are two independent experiments. Statistical significance was determined by the two-tailed unpaired Student's *t*-test. Source data are provided as a Source Data file.

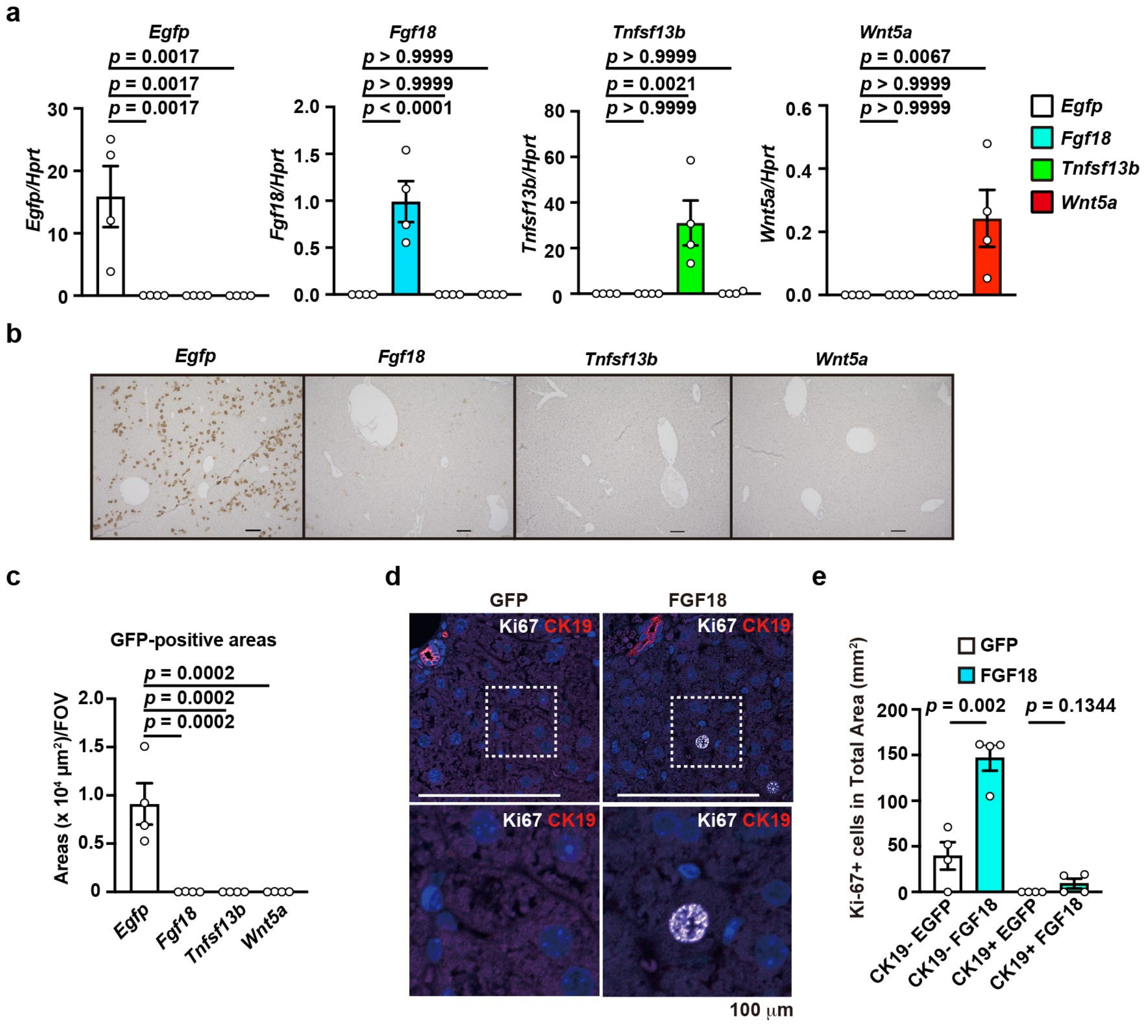

Supplementary Figure 3 | Ki67^{+} cells do not co-express a cholangiocyte marker. Eight-week-old female wild-type mice were injected with the indicated expression vectors by the HTVi method. **a,** The expression of the infused genes in the livers was determined by qPCR. Results are means ± SE (*n* = 4 mice). **b-e**, Liver sections were stained with anti-GFP (**b**) or anti-CK19 and anti-Ki67 antibodies (**d**) (*n* = 4 mice for each condition). Scale bar, 100 μm. GFP^{+} areas (**c**), CK19^{+} Ki-67^{+}, and CK19^{-} Ki67^{+} areas (**e**) were calculated and expressed as positive areas per FOV. Results are means ± SE (*n* = 4 mice) and represent two independent experiments. Significance was determined by the one-way ANOVA with Dunnett's multiple comparison test (**a, c**) or two-tailed unpaired Student's *t*-test (**e**). Source data are provided as a Source Data file.

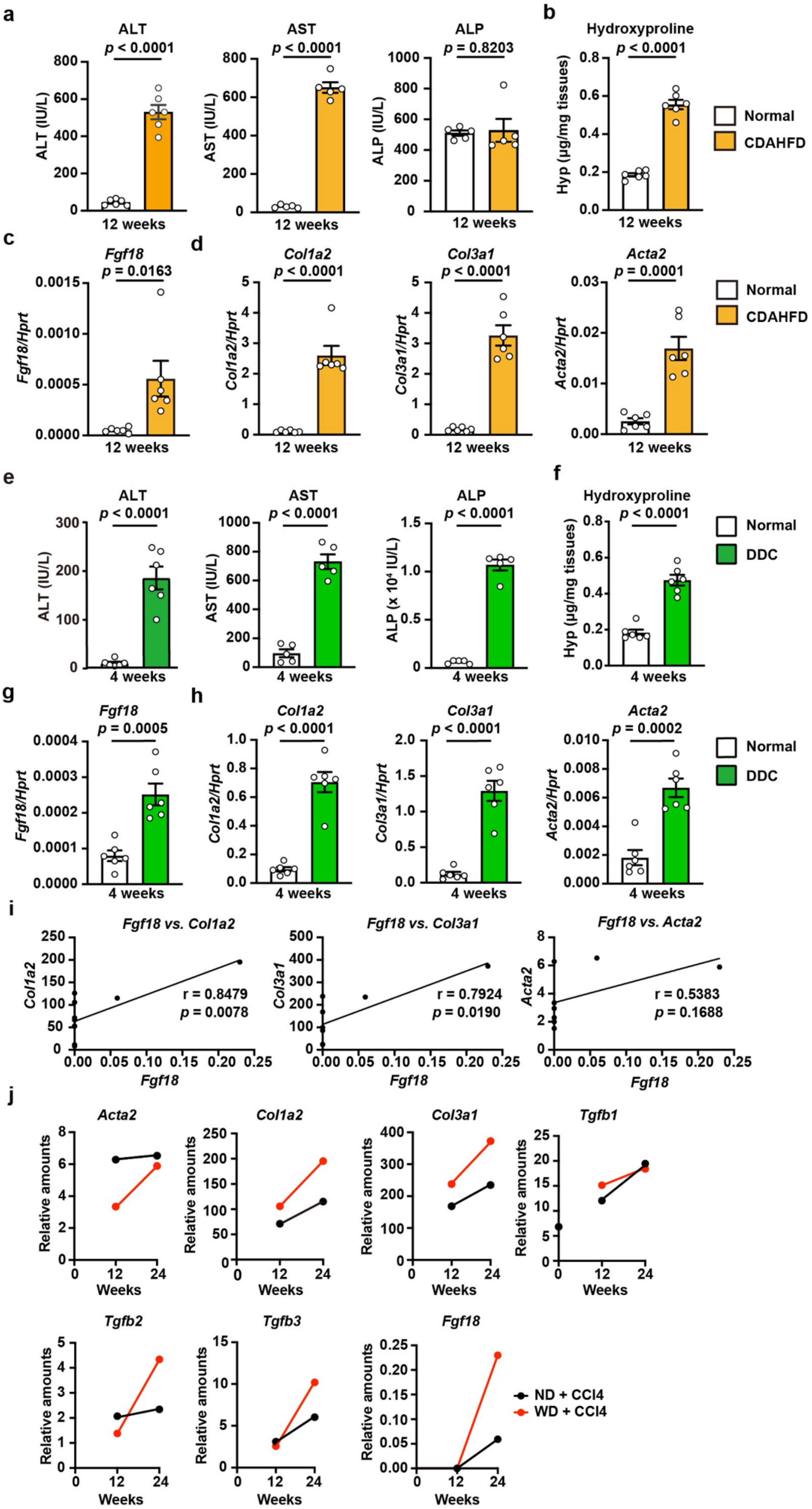
a
ALT
p < 0.0001
ALT (IU/L)
12 weeks
AST
p < 0.0001
AST (IU/L)
12 weeks
ALP
p = 0.8203
ALP (IU/L)
12 weeks
b
Hydroxyproline
p < 0.0001
Hyp (µg/mg tissues)
12 weeks
Normal
CDAHFD
c
Fgf18
p = 0.0163
Fgf18/Hprt
12 weeks
d
Col1a2
p < 0.0001
Col1a2/Hprt
12 weeks
Col3a1
p < 0.0001
Col3a1/Hprt
12 weeks
Acta2
p = 0.0001
Acta2/Hprt
12 weeks
Normal
CDAHFD
e
ALT
p < 0.0001
ALT (IU/L)
4 weeks
AST
p < 0.0001
AST (IU/L)
4 weeks
ALP
p < 0.0001
ALP (x 10⁴ IU/L)
4 weeks
f
Hydroxyproline
p < 0.0001
Hyp (µg/mg tissues)
4 weeks
Normal
DDC
g
Fgf18
p = 0.0005
Fgf18/Hprt
4 weeks
h
Col1a2
p < 0.0001
Col1a2/Hprt
4 weeks
Col3a1
p < 0.0001
Col3a1/Hprt
4 weeks
Acta2
p = 0.0002
Acta2/Hprt
4 weeks
Normal
DDC
i
Fgf18 vs. Col1a2
Col1a2
r = 0.8479
p = 0.0078
Fgf18
Fgf18 vs. Col3a1
Col3a1
r = 0.7924
p = 0.0190
Fgf18
Fgf18 vs. Acta2
Acta2
r = 0.5383
p = 0.1688
Fgf18
j
Acta2
Col1a2
Col3a1
Tgfb1
Tgfb2
Tgfb3
Fgf18
Relative amounts
Weeks
ND + CCl4
WD + CCl4

Supplementary Figure 4 | Expression of *Fgf18* in the livers is elevated in wild-type mice fed various diets. a-h, Eight-week-old wild-type mice were fed the normal diet or the CDAHFD for 12 weeks (**a-d**) or DDC diet for 4 weeks (**e-h**). The serum ALT (n = 6 mice) , AST (n = 5 mice), and ALP (n = 5 mice) concentrations (**a, e**) and the hydroxyproline content (**b, f**) (n = 6 mice) were determined. The expression of *Fgf18* and fibrosis-related genes was determined by qPCR (**c, d, g, h**) (n = 6 mice). Results are mean ± SE. Representative results of two independent experiments are shown. Significance was determined by the two-tailed unpaired Student's t-test. **i**, **j,** The expression of *Fgf18* is upregulated at relatively late stages of a liver fibrosis model. Bulk RNA-seq data (GSE99010) were retrieved from the previous study[1] and reanalysed. Wild-type mice (4 mice for each condition) were injected with olive oil or CCl_4 and fed the normal diet (ND) or Western diet (WD) for the indicated weeks. Extracted mRNAs from the lives of four mice per each condition were pooled and subjected to RNA-seq analysis. The correlation between the expression of *Fgf18* and *Col1a2, Col3a1, or Acta2* was determined by Pearson correlation coefficient analysis. P values were calculated a two-sided test. (**i**). Relative expression of the indicated genes was plotted at the duration of the indicated diet combined with CCl_4 injection (**j**). Source data are provided as a Source Data file.

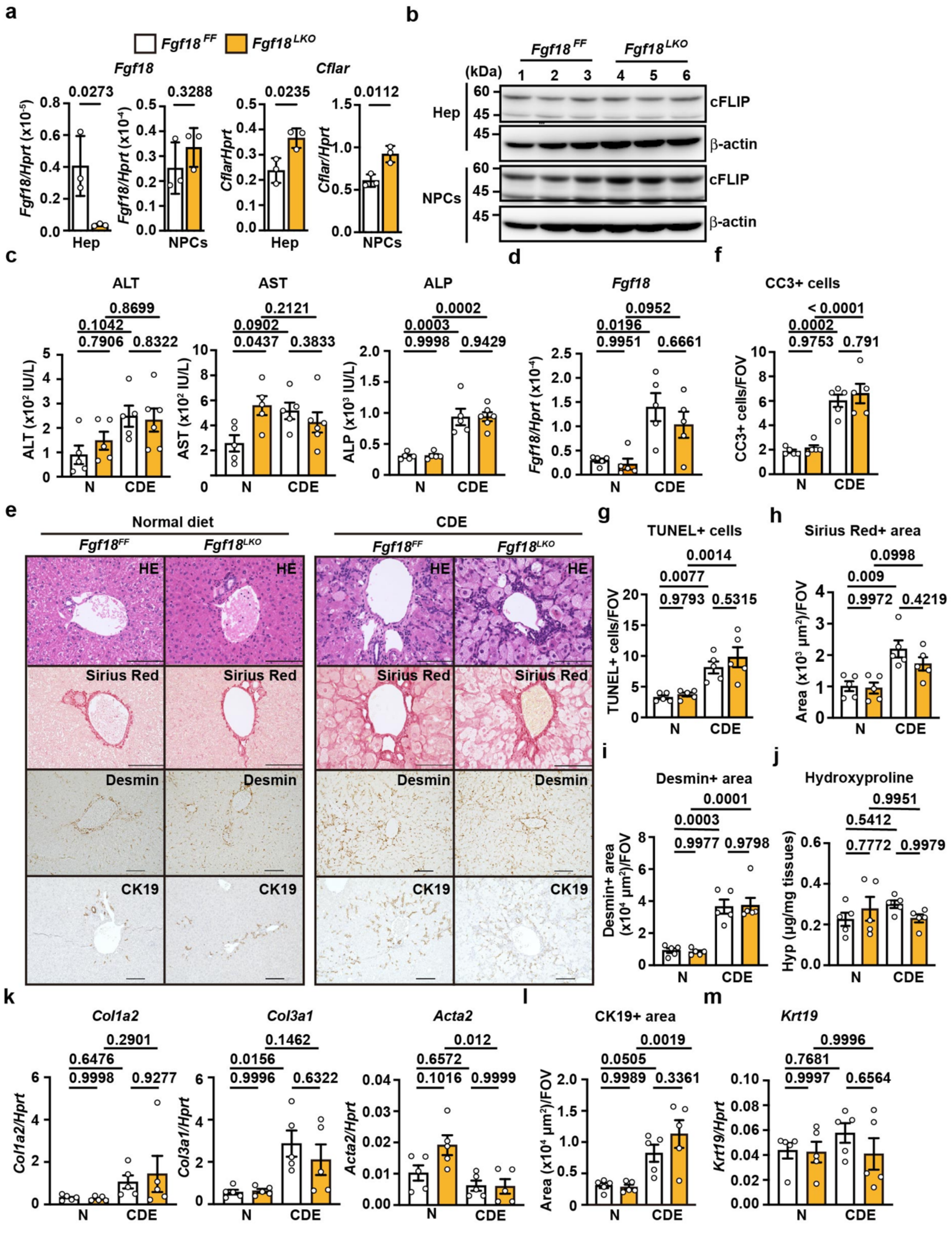

Supplementary Figure 5 | Deletion of *Fgf18* in hepatocytes does not attenuate or exacerbate liver fibrosis in mice fed CDE diet. **a, b**, The expression of *Fgf18* in hepatocytes, but not NPCs, is abolished in *Fgf18*^{LKO} mice. Hepatocytes and NPCs were prepared from 8-week-old *Fgf18*^{FF} and *Fgf18*^{LKO} mice, and the expression of *Fgf18* and *Cflar* was determined by qPCR (**a**). Results are mean ± SE (*n* = 3 mice). The expression

of cFLIP in hepatocytes and NPCs was analyzed by Western blotting (**b**). Numbers indicate an individual mouse. Results are representative of two independent experiments **c - m**, Eight-week-old female *Fgf18*^{FF} and *Fgf18*^{LKO} mice were fed the normal diet (N) or CDE for 4 weeks. The serum ALT, AST, and ALP concentrations were determined (**c**). Results are mean ± SE (n = 5 mice). The expression of *Fgf18* in the livers was determined by qPCR (**d**). Results are mean ± SE (n = 5 mice). Liver sections from mice of the indicated genotypes were stained with H&E, anti-CC3 antibody, TUNEL, Sirius Red, and anti-CK19 antibody (**e**) (n = 5). Scale bar, 100 µm. The numbers of CC3^{+} (**f**), and TUNEL^{+} (**g**) cells were counted as expressed as in Fig. 1d. The Sirius Red^{+} (**h**), desmin^{+} (**i**), and CK19^{+} (**l**) areas were quantified and expressed as in Fig. 1f. Results are mean ± SE (n = 5 mice). The hydroxyproline content of the livers was determined (**j**). Results are mean ± SE (n = 5 mice). The expression of the indicated genes in the livers was determined by qPCR (**k, m**). Results are mean ± SE (n = 5 mice). Pooled results of four independent experiments are shown. Statistical significance was determined by the two-tailed unpaired Student's *t*-test (**a**) or two-way ANOVA with Tukey's multiple comparison test (**c, d, f-m**). Source data are provided as a Source Data file.

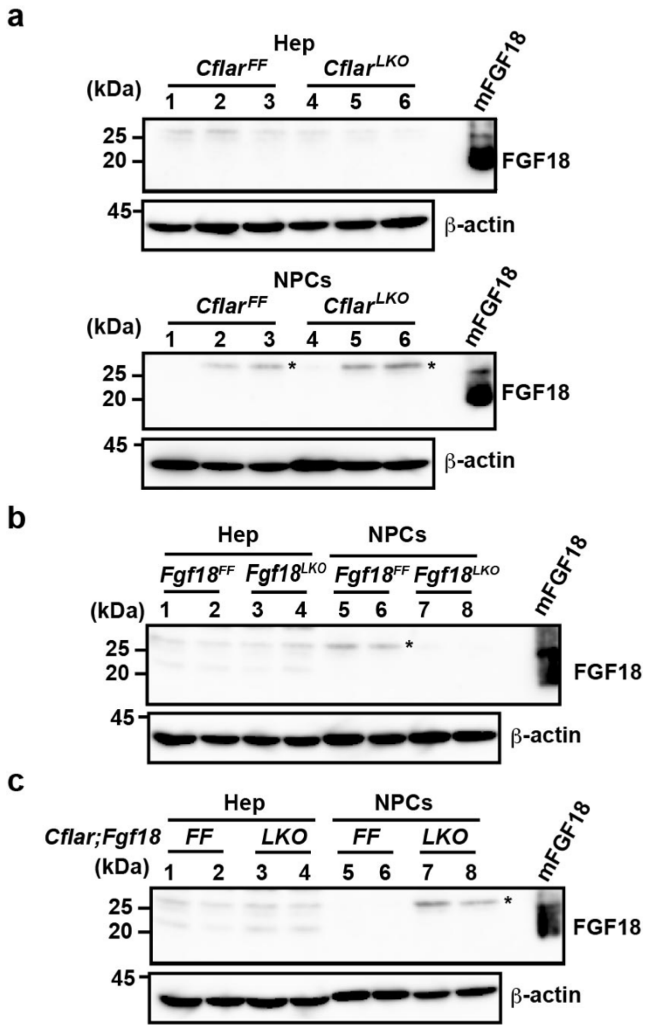

Supplementary Figure 6 | Protein expression of FGF18 is not detected in hepatocytes or NPCs from mice of the indicated genotypes. Hepatocytes and NPCs were isolated from mice of the indicated genotypes [$n = 3$ for *Cflar*^{FF} and *Cflar*^{LKO} mice (**a**); $n = 2$ for *Fgf18*^{FF} and *Fgf18*^{LKO} mice (**b**); $n = 2$ for *Cflar*^{FF}*;Fgf18*^{FF} and *Cflar*^{LKO}*;Fgf18*^{LKO} mice (**c**)] and the expression of FGF18 at protein levels was analyzed by Western blotting. Each number indicates an individual mouse. mFGF18 indicates recombinant murine FGF18 generated in *E. coli* in house. Asterisks indicate cross-reacted bands. Results are representative of two independent experiments. Source data are provided as a Source Data file.

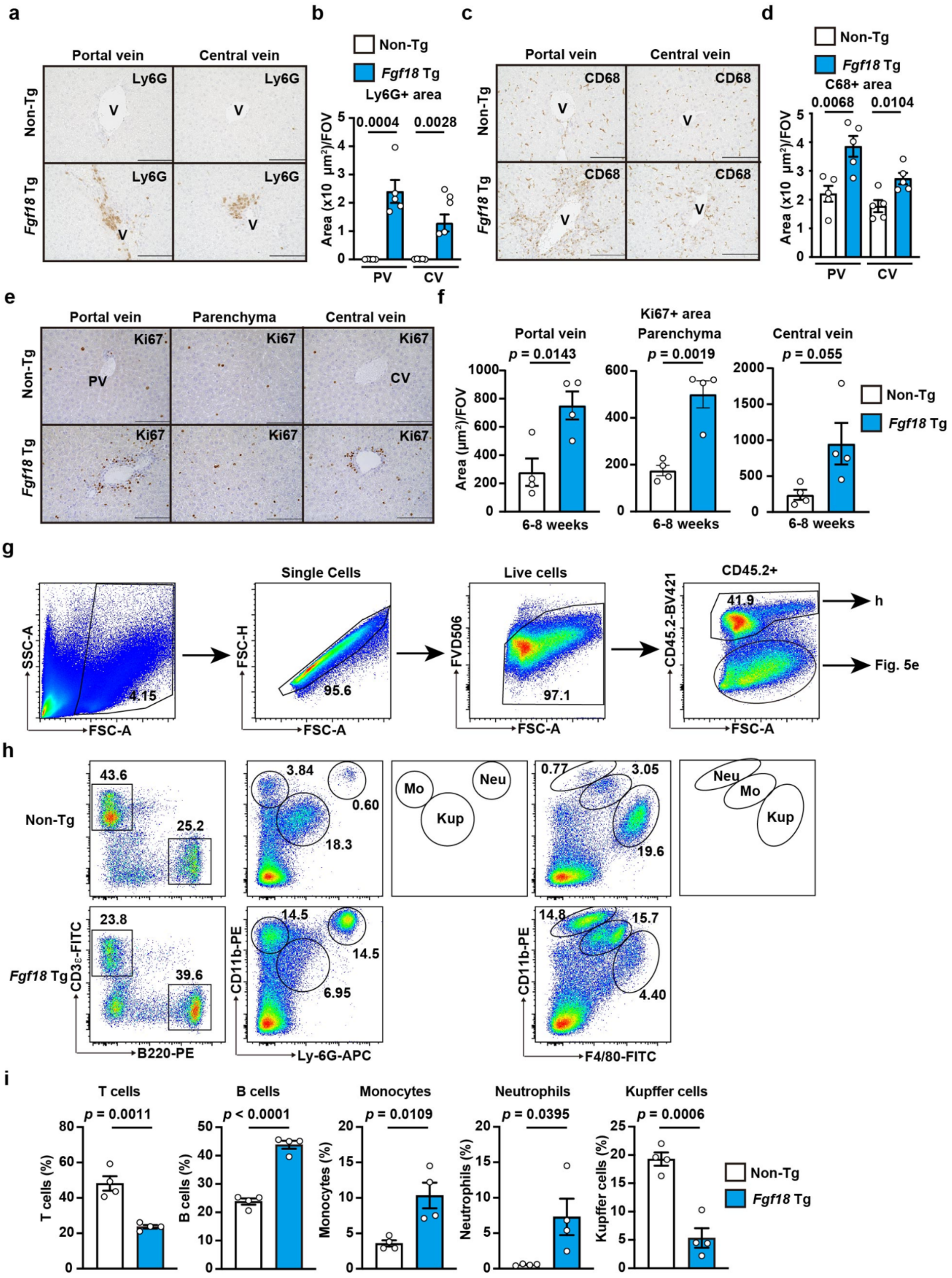
a
Portal vein
Central vein
Non-Tg
Fgf18 Tg
Ly6G
V
b
Non-Tg
Fgf18 Tg
Ly6G+ area
0.0004
0.0028
Area (x10 μm²)/FOV
PV
CV
c
Portal vein
Central vein
CD68
d
Non-Tg
Fgf18 Tg
C68+ area
0.0068
0.0104
e
Portal vein
Parenchyma
Central vein
Ki67
PV
CV
f
Ki67+ area
Portal vein
p = 0.0143
Parenchyma
p = 0.0019
Central vein
p = 0.055
Area (μm²)/FOV
6-8 weeks
g
SSC-A
FSC-A
4.15
Single Cells
FSC-H
95.6
Live cells
FVD506
97.1
CD45.2+
CD45.2-BV421
41.9
h
Fig. 5e
CD3ε-FITC
B220-PE
CD11b-PE
Ly-6G-APC
F4/80-FITC
Mo
Neu
Kup
43.6
25.2
3.84
0.60
18.3
0.77
3.05
19.6
23.8
39.6
14.5
14.5
6.95
14.8
15.7
4.40
i
T cells
p = 0.0011
T cells (%)
B cells
p < 0.0001
B cells (%)
Monocytes
p = 0.0109
Monocytes (%)
Neutrophils
p = 0.0395
Neutrophils (%)
Kupffer cells
p = 0.0006
Kupffer cells (%)

Supplementary Figure 7 | Characterization of cells in the livers of *Fgf18* Tg mice. **a-f**, Liver sections from 6 to 8-week-old non-Tg and *Fgf18* Tg mice were stained with anti-Ly6G (**a**), anti-CD68 (**c**), or anti-Ki67 (**e**) antibodies (*n* = 5 mice). Scale bar, 100 μm. Ly6G-, CD68-, and Ki67-positive areas were quantified and expressed as in Fig. 1c (**b, d, f**). Results are mean ± SE (n = 4 mice). **g-i**, Liver non-parenchymal cells were prepared from 6- to 8-week-old non-Tg and *Fgf18* Tg mice and analyzed by flow cytometry. Gating strategy (**g**). The percentages of cell populations surrounded by circles are shown (**h**). Mo, monocytes; Neu, neutrophils; Kup, Kupffer cells. The percentages of the indicated cells gated on $CD45^+$ cells were calculated and are shown (**i**). Results are mean ± SE (*n* = 4 mice). Statistical significance was determined by the two-tailed unpaired Student's *t*-test. Source data are provided as a Source Data file.

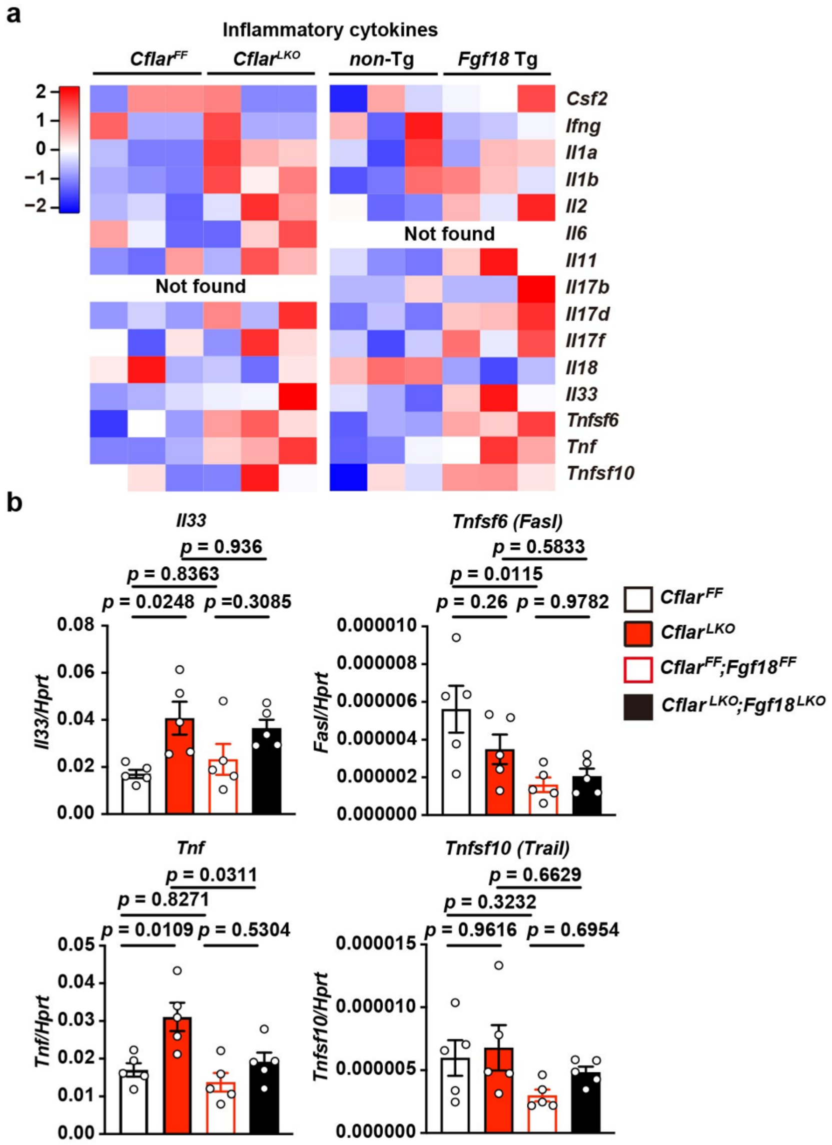

Supplementary Figure 8 | Overexpression of *Fgf18* induces the expression of inflammatory cytokine genes. a, Heatmap showing Z-score scaled expression levels of inflammatory cytokine genes (n = 3 mice). The RNA-seq data of the whole livers from mice of the indicated genotypes in Figs. 2 and 5 were analyzed. "Not found" indicates the genes in the livers of *Cflar^{LKO}* and *Fgf18* Tg mice that were not upregulated more than two fold compared to those in *Cflar^{FF}* and non-Tg mice, respectively. **b**, Deletion of *Fgf18* in hepatocytes reduces the expression of *Tnf,* but not other cytokine genes. The expression of the indicated genes in the livers of mice of the indicated genotypes fed the CDE diet for 4 weeks was determined by qPCR. Results are mean ± SE (n = 5 mice). Pooled results of four independent experiments are shown. Statistical significance was determined by the one-way ANOVA with Tukey's multiple comparison. Source data are provided as a Source Data file.

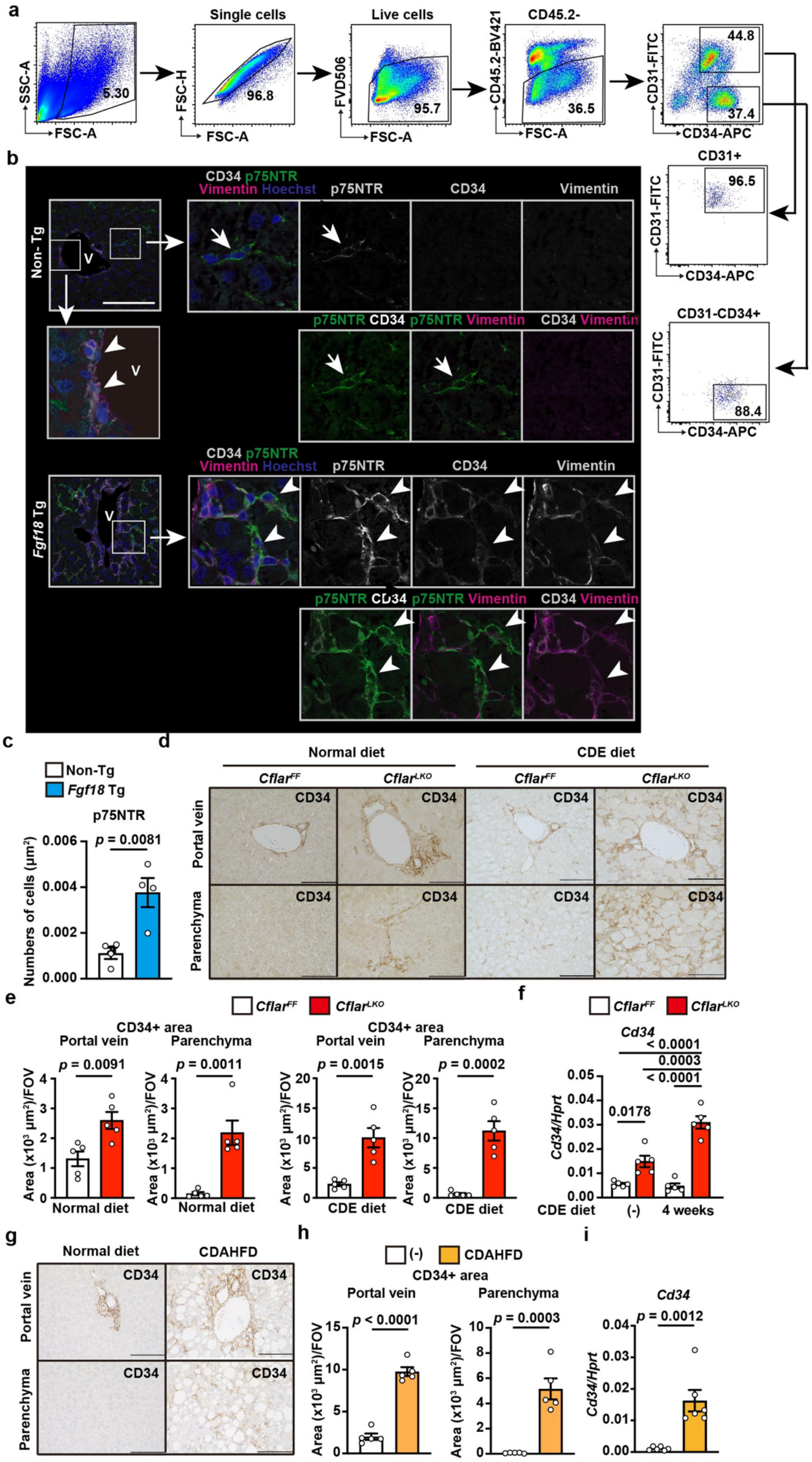
a
SSC-A
FSC-A
5.30
Single cells
FSC-H
96.8
Live cells
FVD506
95.7
CD45.2-
CD45.2-BV421
36.5
CD31-FITC
CD34-APC
44.8
37.4
CD31+
96.5
CD31-CD34+
88.4
b
Non- Tg
Fgf18 Tg
CD34 p75NTR Vimentin Hoechst
p75NTR
CD34
Vimentin
p75NTR CD34
p75NTR Vimentin
CD34 Vimentin
V
c
Non-Tg
Fgf18 Tg
p75NTR
Numbers of cells (μm²)
p = 0.0081
d
Normal diet
CDE diet
Cflar^FF
Cflar^LKO
Portal vein
Parenchyma
CD34
e
CD34+ area
Portal vein
Parenchyma
Area (x10³ μm²)/FOV
p = 0.0091
p = 0.0011
p = 0.0015
p = 0.0002
Normal diet
CDE diet
f
Cd34
Cd34/Hprt
< 0.0001
0.0003
< 0.0001
0.0178
CDE diet
(-)
4 weeks
g
Normal diet
CDAHFD
h
(-)
CDAHFD
p < 0.0001
p = 0.0003
i
p = 0.0012

Supplementary Figure 9 | The number of CD34$^+$ cells are increased in the livers of *Fgf18* Tg mice. a, Cell sorting strategy for CD31$^+$ cells and CD31$^-$CD34$^+$ cells. Liver non-parenchymal cells were prepared from 6 to 8-week-old *Fgf18* Tg mice and analyzed by flow cytometry as in Supplementary Fig. 7g. Results are representative of four to five independent experiments. The percentage of the cell populations in each panel is shown. **b**, **c**, Liver sections of 8- to 10-week-old non-Tg and *Fgf18* Tg mice were stained with anti-CD34, anti-vimentin, or anti-p75NTR antibodies (n = 4 mice) (**b**). The right panels are enlarged images of the left white box. Arrows indicate cells expressing only p75NTR; Arrowheads indicate cells expressing p75NTR, CD34, and vimentin. Scale bars, 100 µm. p75NTR$^+$ cells were counted and are expressed as the numbers of positive cells per field (**c**). Results are mean ± SE (n = 4 mice). **d**, **e**, Mice were treated as in Fig. 1, and liver sections were stained with anti-CD34 antibody (n = 5 mice) (**d**). Scale bar, 100 µm. CD34$^+$ areas were quantified and expressed as areas of the field of view (FOV) (**e**). Results are mean ± SE (n = 5 mice). **f**, Kinetics of the expression of *Cd34* in the livers of eight-week-old female mice before and after CDE diet feeding for 4 weeks. The expression of *Cd34* was determined by qPCR. Results are mean ± SE (n = 5 mice). Pooled results from four independent experiments are shown. **g-i**, Eight-week-old wild-type mice fed the normal diet or the CDAHFD for 12 weeks were analyzed as in **d, e**. Results are mean ± SE (n = 5 mice). The expression of *Cd34* was determined by qPCR (**i**). Results are mean ± SE (n = 5 mice). Representative results from two independent experiments. Statistical significance was determined by the two-tailed unpaired Student's t-test (**c, e, h, i**) or two-way ANOVA with Tukey's multiple comparison test (**f**). Source data are provided as a Source Data file.

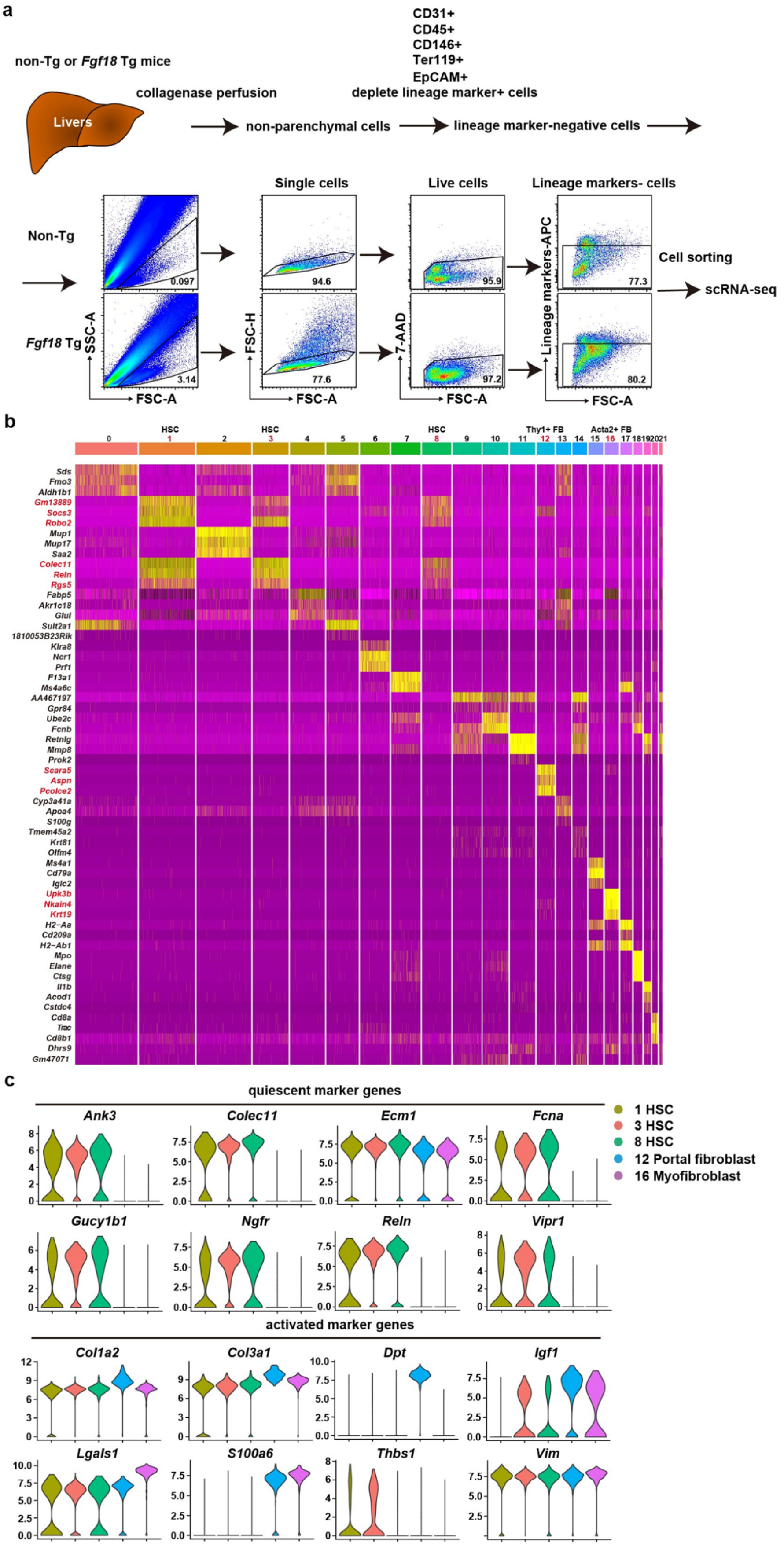
a
non-Tg or Fgf18 Tg mice
Livers
collagenase perfusion
non-parenchymal cells
CD31+
CD45+
CD146+
Ter119+
EpCAM+
deplete lineage marker+ cells
lineage marker-negative cells
Single cells
Live cells
Lineage markers- cells
Non-Tg
Fgf18 Tg
SSC-A
FSC-A
FSC-H
7-AAD
Lineage markers-APC
Cell sorting
scRNA-seq
b
HSC
Thy1+ FB
Acta2+ FB
c
quiescent marker genes
activated marker genes
1 HSC
3 HSC
8 HSC
12 Portal fibroblast
16 Myofibroblast

Supplementary Figure 10 | Signature genes in each cluster. a, Cell sorting strategy for scRNA-seq. Liver non-parenchymal cells were prepared from 6- to 8-week-old non-Tg and *Fgf18* Tg mice and then lineage marker-positive cells were depleted using beads-conjugated antibodies against the indicated lineage markers. After depletion, lineage marker-negative cells were further sorted by a cell sorter and subjected to scRNA-seq. **b**, Heatmap depicting the top three representative genes (on the left side) for each clusters. Representative genes for HSC (*Lrat*$^{+}$), portal fibroblast (*Thy1*$^{+}$ FB), and myofibroblast (*Acta2*$^{+}$ FB) clusters are written in red. FB, fibroblast. **c**, Violin plot showing the expression levels of marker genes for quiescent and activated HSC, portal fibroblast, and myofibroblast pooled from non-Tg and *Fgf18* Tg mice.

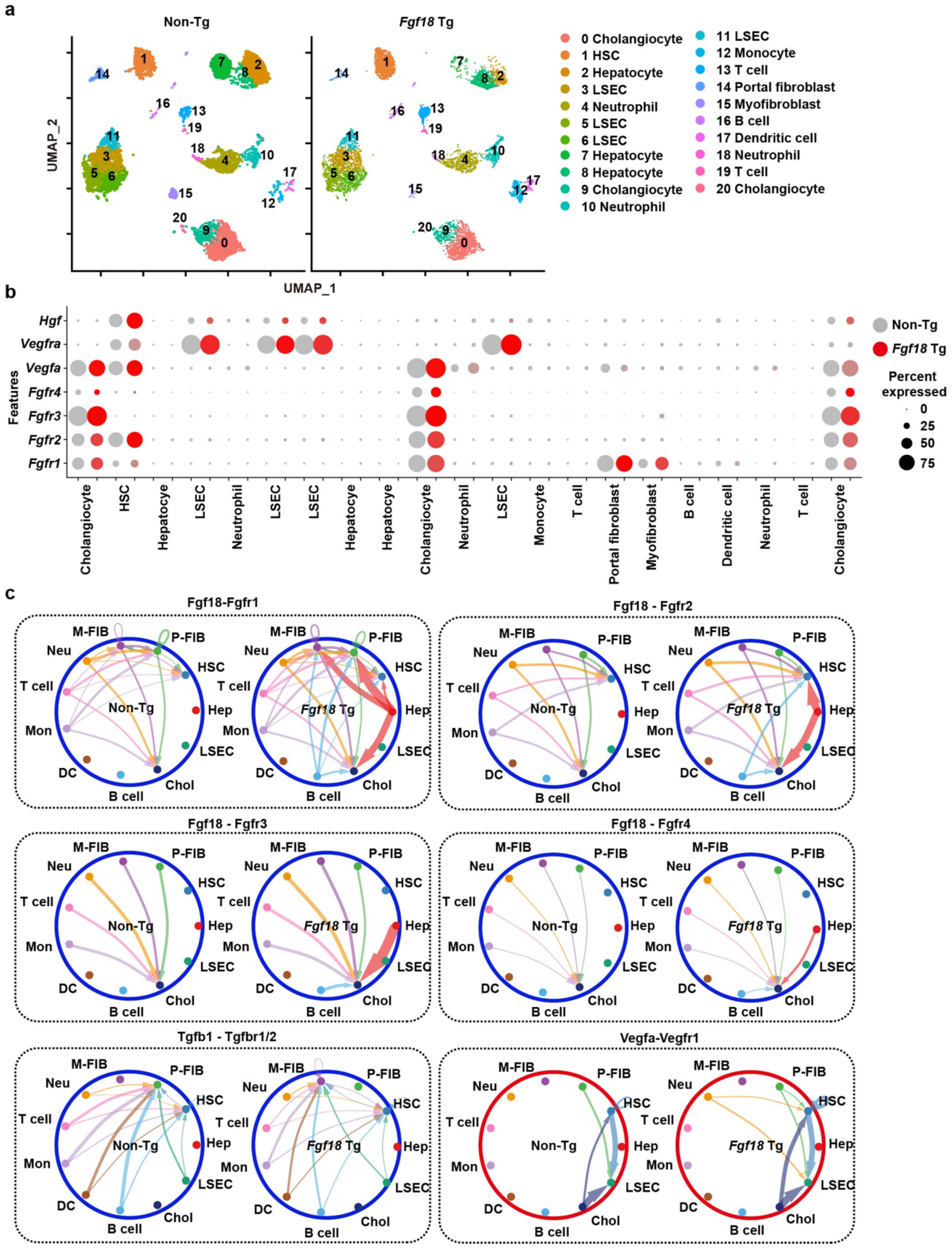
a
Non-Tg
Fgf18 Tg
0 Cholangiocyte
1 HSC
2 Hepatocyte
3 LSEC
4 Neutrophil
5 LSEC
6 LSEC
7 Hepatocyte
8 Hepatocyte
9 Cholangiocyte
10 Neutrophil
11 LSEC
12 Monocyte
13 T cell
14 Portal fibroblast
15 Myofibroblast
16 B cell
17 Dendritic cell
18 Neutrophil
19 T cell
20 Cholangiocyte
UMAP_1
UMAP_2
b
Hgf
Vegfra
Vegfa
Fgfr4
Fgfr3
Fgfr2
Fgfr1
Features
Non-Tg
Fgf18 Tg
Percent expressed
0
25
50
75
c
Fgf18-Fgfr1
Fgf18 - Fgfr2
Fgf18 - Fgfr3
Fgf18 - Fgfr4
Tgfb1 - Tgfbr1/2
Vegfa-Vegfr1
M-FIB
P-FIB
HSC
Hep
LSEC
Chol
B cell
DC
Mon
T cell
Neu

Supplementary Figure 11 | Cell-cell communication in fibrosis and angiogenesis. a, UMAP of combined clusters of scRNA-seq data from non-Tg and *Fgf18* Tg mice with cholangiocyte and LSEC scRNA-seq clusters from untreated wild-type mice. scRNA-seq data of wild-type mice were retrieved from the previous study[2]. Note the combined UMAP contains clusters of cholangiocyte (0, 9, 20) and LSEC (3, 5, 6, 11). **b,** Circle plots showing the expression levels of the indicated genes and percentages of cell expressing the indicated genes from non-Tg and *Fgf18* Tg mice. Color intensities and circle sizes indicate the expression levels and percentages of expressing cells of the indicated genes, respectively. **c,** Communication networks among HSC, fibroblast, and other types of cell. Cell-cell communication networks were analyzed by CellChat. All the significant ligand-receptor pairs that contribute to sending signals from one cell to another cell. Edge width represents the communication probability. Of note, weak *Fgf18* signals appeared to originate from various types of cells, which may be caused by readthrough transcripts of *Fgf18* expressed in various tissues from Fgf18 Tg mice. M-FIB, myofibroblast; P-FIB, portal fibroblast; HSC, hepatic stellate cell; Hep, hepatocyte; LSEC, liver sinusoidal cell; Chol, cholangiocyte; DC, dendritic cell; Mo, monocyte; Neu, neutrophil.

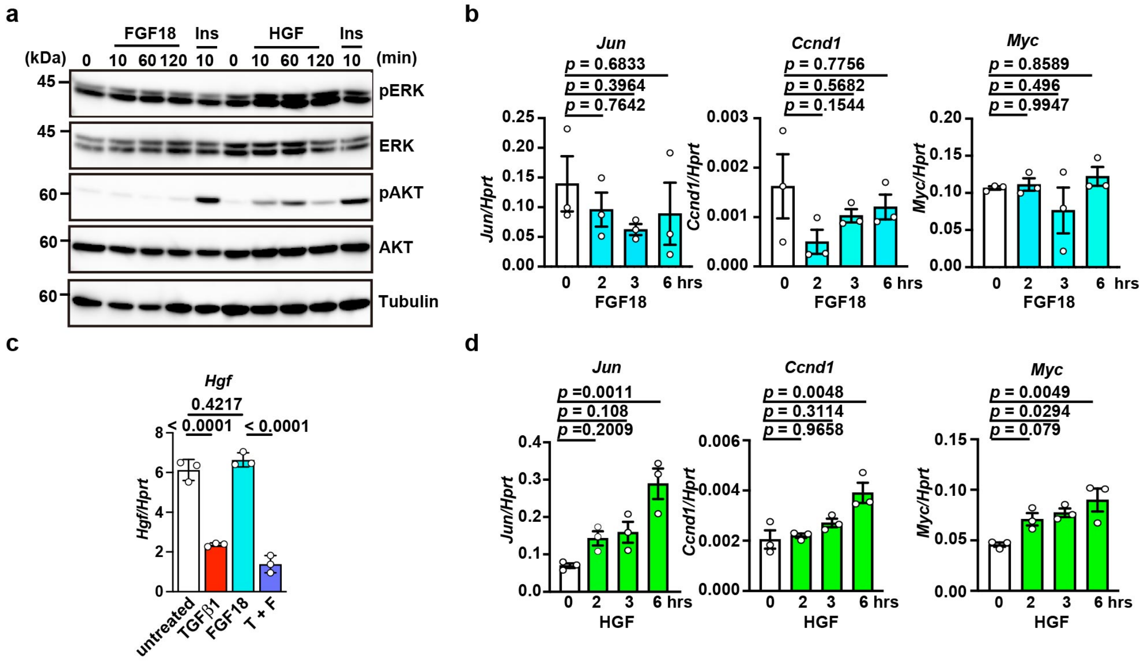

Supplementary Figure 12 | HGF, but not FGF18, induces signals into hepatocytes. a, b, d, Primary hepatocytes prepared from eight-week-old wild-type mice were stimulated with FGF18 (100 ng/mL), insulin (10 µg/mL), or HGF (100 ng/mL) for the indicated times. Phosphorylated and total ERK, and phosphorylated and total AKT were analyzed by immunoblotting with the indicated antibodies (**a**). The expression of *Jun, Ccnd1*, and *Myc* was determined by qPCR (**b, d**). Results are mean ± SD of triplicate samples. **c**, Isolated HSCs were stimulated with TGFβ1 (1 ng/mL), FGF18 (100 ng/mL), or TGFβ1 (1 ng/mL) + FGF18 (100 ng/mL) for 24 hours. The expression of *Hgf* was analyzed by qPCR. Results are mean ± SD of triplicate samples. All results are representative of two independent experiments. Statistical significance was determined by one-way ANOVA with Dunnett's multiple comparison test (**b, d**) or one-way ANOVA with Tukey's multiple comparison test (**c**). Source data are provided as a Source Data file.

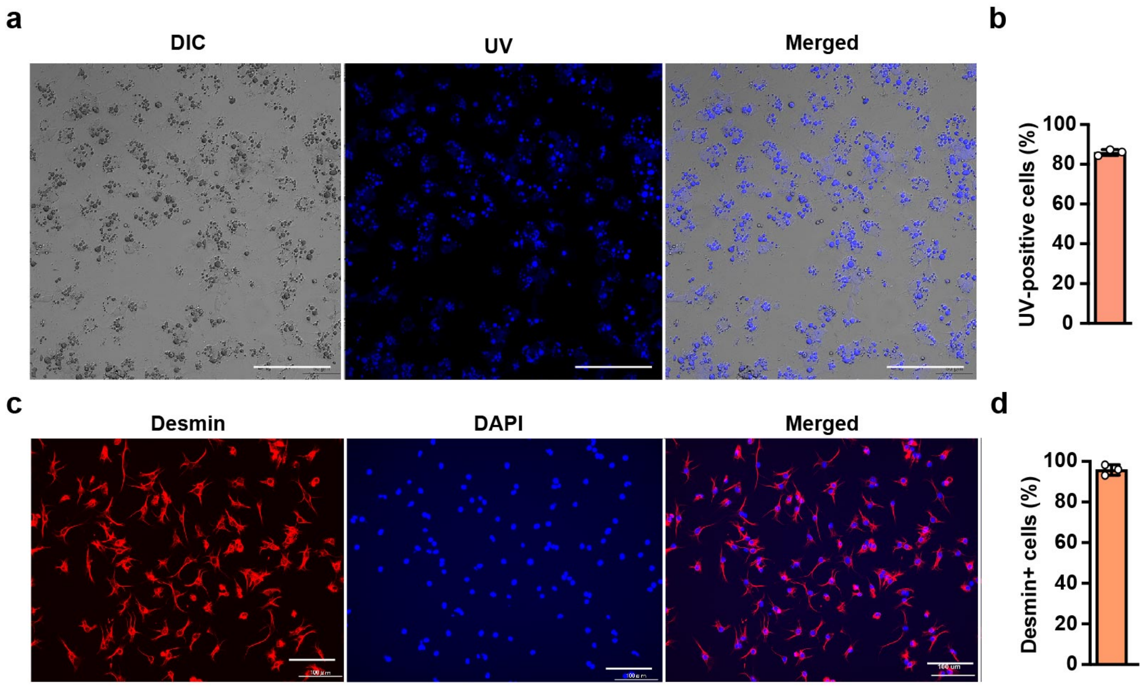

Supplementary Figure 13 | The percentages of HSCs isolated using the Nycodenz gravity gradient centrifugation method. a - d, HSCs were isolated as in the Methods and plated onto the dish. After removing nonadherent cells, the purity was determined by UV (**a**) or anti-desmin antibody staining (**c**). The percentages of UV^{+} cells among all cells based on differential interference contrast (DIC) (**b**), or $desmin^{+}$ cells among DAPI-positive cells (**d**) were determined by calculating three randomly picked up fields. Scale bars, 100 μm. Results are mean ± SD of triplicate samples. Results are representative of two independent experiments. Source data are provided as a Source Data file.

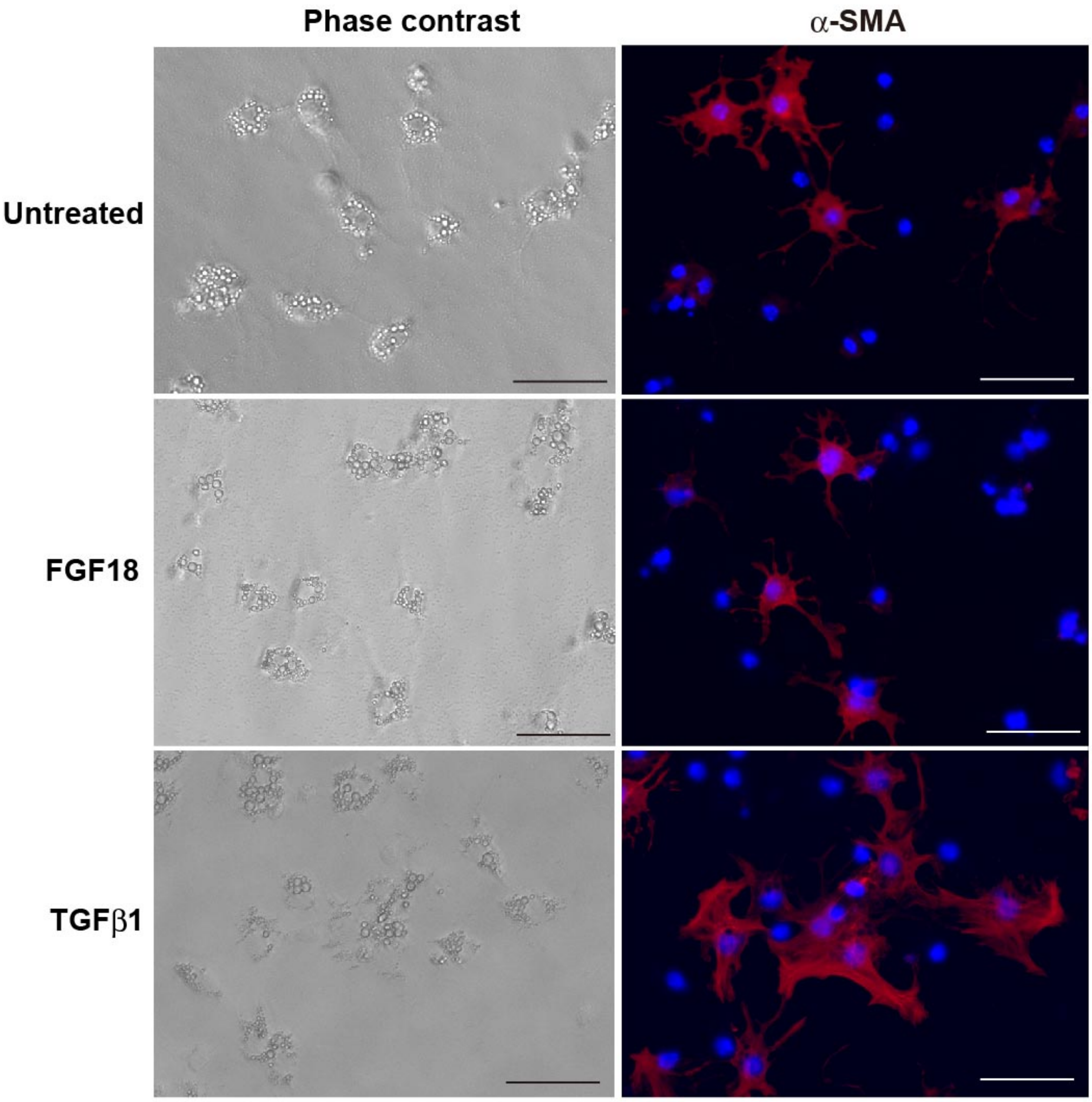

Supplementary Figure 14 | FGF18 does not induce morphological changes of HSCs. HSCs were plated onto the dish and then untreated or simulated with FGF18 (100 ng/mL) or TGFβ1 (1 ng/mL) for 24 hours. Cells were fixed, permeabilized, and stained with anti-α-SMA antibodies and DAPI, followed by visualization with Alexa 647-conjugated secondary antibody. Results are representative of two independent experiments. Scale bars, 50 μm.

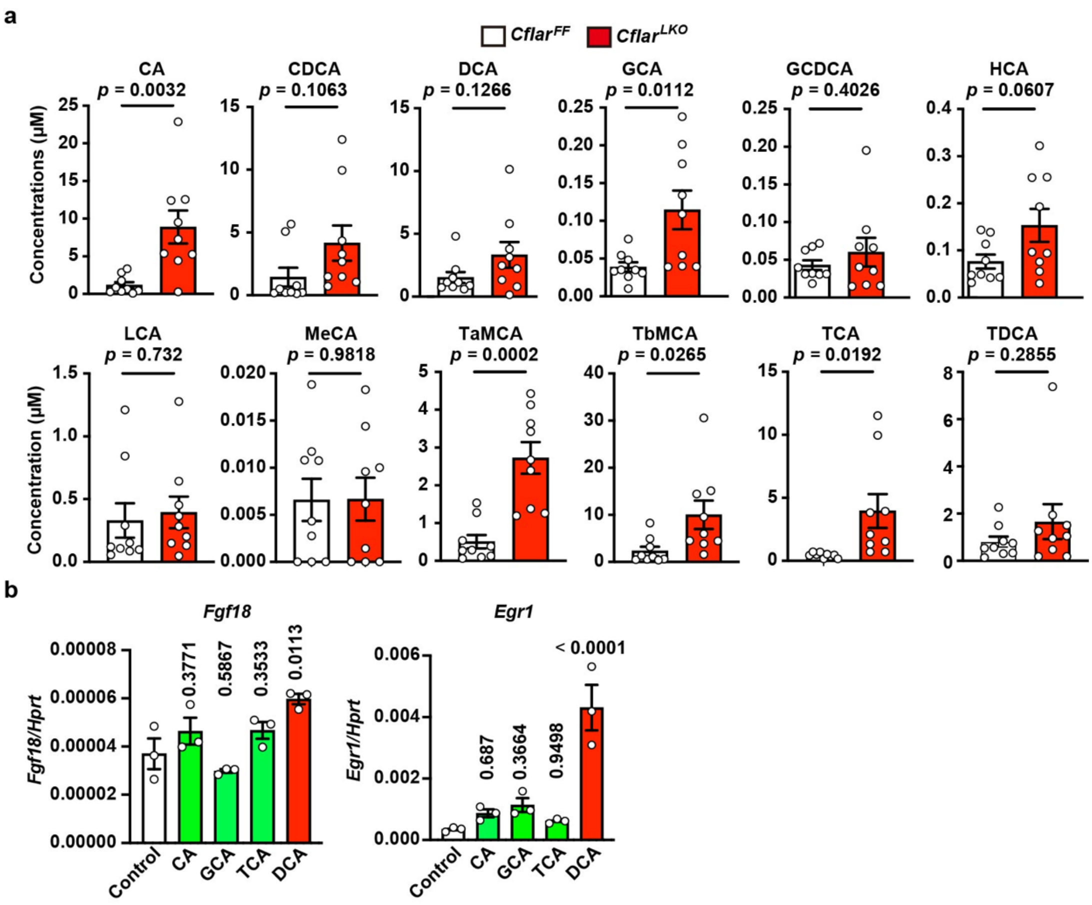

Supplementary Figure 15 | Elevated bile acids are not responsible for upregulation of *Fgf18*. a, Eight-week-old female *Cflar*^{FF} and *Cflar*^{LKO} mice were fed the CDE diet for 4 weeks. Serum bile acid levels were determined by LC-MS analysis and are expressed as their concentrations (μM). Results are mean ± SE (n = 9 mice). Pooled results from two to three independent experiments are shown. CA, cholic acid; CDCA, chenodeoxycholic acid; DCA, deoxycholic acid; GCA, glycocholic acid; GCDCA, glycochenodeoxycholic acid; HCA, hyocholic acid; LCA, lithocholic acid; MeCA, methylcholic acid; TaMCA, tauro-α-muricholic acid; TbMCA, tauro-β-muricholic acid; TCA, taurocholic acid; and TDCA, taurodeoxycholic acid. **b**, Bile acids do not induce elevation of *Fgf18* mRNA. Primary hepatocytes from 8-week-old wild-type mice were prepared and stimulated with control or 100 μM of the indicated bile acids for 2 hours. The expression of *Fgf18* or *Egr1* was determined by qPCR. *Egr1* is a positive control for DCA-induced genes. Results are mean ± SD of triplicate samples. Results are representative of two independent experiments. Statistical significance was determined by the two-tailed unpaired Student's *t*-test (**a**) or one-way ANOVA with Dunnett's multiple comparison test (**b**). Source data are provided as a Source Data file.

Supplementary Table 1. Expression of *Fgf18* is elevated in mice fed the normal or Western diet treated with CCl_4 for 24 weeks.

	12 weeks				24 weeks			
	Normal diet		Western diet		Normal diet		Western diet	
Gene symbols	oil	CCl_4	oil	CCl_4	oil	CCl_4	oil	CCl_4
Acta2	2.00	6.28	2.95	3.34	2.29	6.53	1.52	5.89
Col1a2	11.40	71.12	52.51	105.81	8.33	115.18	53.06	195.26
Col3a1	23.51	168.65	99.17	237.96	24.25	234.79	85.91	372.47
Fgf18	0.00	0.00	0.00	0.00	0.00	0.06	0.00	0.23
Tgfb1	6.83	12.05	11.07	15.13	7.50	19.47	10.23	18.41
Tgfb2	0.89	2.07	1.03	1.38	0.32	2.35	0.85	4.34
Tgfb3	1.60	3.10	2.31	2.57	1.42	6.03	2.71	10.20

Reanalysis of GSE99010. Wild-type mice (4 mice for each condition) were fed the normal diet (ND) or Western diet (WD) combined with olive oil (oil) or CCl_4 injection for the indicated weeks. RNAs extracted from the livers of four mice under each condition were pooled, and pooled RNAs were analyzed by RNA-seq. Relative mRNA expression levels of the indicated genes at each condition are shown.

Supplementary Table 2. Signature genes for each cluster.

Clusters in Figure 6		
Cluster No.	Cell type	Signature genes
0	Hepatocyte	*Alb*
1	HSC	*Lrat*
2	Hepatocyte	*Alb*
3	HSC	*Lrat*
4	Hepatocyte	*Alb*
5	Hepatocyte	*Alb*
6	T cell	*Lck*
7	Monocyte	*Cd14, Cx3cr1*
8	HSC	*Lrat*
9	Neutrophil	*Ly6g, Itgam*
10	Neutrophil	*Ly6g, Itgam*
11	Neutrophil	*Ly6g, Itgam*
12	Portal fibroblast	*Thy1*
13	Hepatocyte	*Alb*
14	Neutrophil	*Ly6g, Itgam*
15	B cell	*Cd19*
16	Myofibroblast	*Acta2*
17	Dendritic cell	*Itgax*
18	Neutrophil	*Ly6g, Itgam*
19	Neutrophil	*Ly6g, Itgam*
20	T cell	*Lck*
21	Neutrophil	*Ly6g, Itgam*

Clusters in Supplementary Figure 9		
Cluster No.	Cell type	Signature genes
0	HSC	*Lrat*
1	Cholangiocyte	*Sox9, Igfbp6*
2	Hepatocyte	*Alb*
3	HSC	*Lrat*
4	Hepatocyte	*Alb*
5	LSEC	*Cdh5, Cldn5*
6	Hepatocyte	*Alb*
7	LSEC	*Cdh5, Cldn5*
8	Neutrophil	*Ly6g, Itgam*
9	Neutrophil	*Ly6g, Itgam*
10	Hepatocyte	*Alb*
11	Neutrophil	*Ly6g, Itgam*
12	T cell	*Lck*
13	Neutrophil	*Ly6g, Itgam*
14	Portal fibroblast	*Thy1*
15	LSEC	*Cdh5, Cldn5*
16	Cholangiocyte	*Sox9, Igfbp6*
17	B cell	*Cd19*
18	Myofibroblast	*Acta2*
19	Dendritic cell	*Itgax*
20	Neutrophil	*Ly6g, Itgam*
21	Myofibroblast	*Acta2*
22	Neutrophil	*Ly6g, Itgam*
23	T cell	*Lck*
24	HSC	*Lrat*
25	Myofibroblast	*Acta2*
26	HSC	*Lrat*

Signature genes to define each cluster from scRNA-seq analysis are shown.

Supplementary Table 3. Characteristics of the patients who underwent liver biopsy

Gender	Male	Female	Total
Number of patients	8	15	23
Age (mean ± SD)	52.0 ± 17.1	53.5 ± 11.5	53.0 ± 13.3
AST (IU/L) (mean ± SD)	73.3 ± 47.5	66.7 ± 40.9	69.0 ± 42.4
ALT (IU/L) (mean ± SD)	111.3 ± 91.9	92.4 ± 74.7	99.0 ± 79.6
γ-GTP (IU/L) (mean ± SD)	127.3 ± 115.6	156.5 ± 181.7	146.3 ± 159.6
T-Cho (mg/dl) (mean ± SD)	157.9 ± 18.9	205.5 ± 39.1	188.9 ± 40.3
MASH	3	4	7
Autoimmune hepatitis	1	2	3
IPH	0	1	1
Alcoholic cirrhosis	1	1	2
Chronic hepatitis	1	1	2
PBC	0	3	3
Others	2	3	5

MASH, metabolic dysfunction-associated steatohepatitis; IPH, idiopathic portal hypertension; PBC, primary biliary cholangitis.

Supplementary Table 4. Primers used in this study.

Murine primers:

Acta2:	Fwd	5’-CTGACAGAGGCACCACTGAA-3’
	Rev	5’- CATCTCCAGAGTCCAGCACA-3’
Ccnd1:	Fwd	5’-CAGAAGTGCGAAGAGGAGGTC-3’
	Rev	5’-TCATCTTAGAGGCCACGAACAT-3’
Cd34:	Fwd	5’-AAGGCTGGGTGAAGACCCTTA-3’
	Rev	5’-TGAATGGCCGTTTCTGGAAGT-3’
Cflar:	Fwd	5’- GGTGGAAGAGTGTCTTGATGAAG-3’
	Rev	5’- CCCTGACGTTAGGTGCAGC-3’
Col1a2:	Fwd	5’-CAGAACATCACCTACCACTGCAA-3’
	Rev:	5’-TTCAACATCGTTGGAACCCTG-3’
Col3a1:	Fwd	5’-CTGTAACATGGAAACTGGGGAAA-3’
	Rev	5’-CCATAGCTGAACTGAAAACCACC-3’
Egr1:	Fwd	5’-TCGGCTCCTTTCCTCACTCA-3’
	Rev	5’-CTCATAGGGTTGTTCGCTCGG-3’
Fgf7:	Fwd	5’-TTTGGAAAGAGCGACGACTT-3’
	Rev	5’-GGCAGGATCCGTGTCAGTAT-3’
Fgf18:	Fwd	5’-CCTGCACTTGCCTGTGTTTAC-3’
	Rev	5’-TGCTTCCGACTCACATCATCT-3’
Fgfr1:	Fwd	5’-TAATACCACCGACAAGGAAATGG-3’
	Rev	5’-TGATGGGAGAGTCCGATAGAGT-3’
Fgfr2:	Fwd	5’-AATCTCCCAACCAGAAGCGTA-3’
	Rev	5’-CTCCCCAATAAGCACTGTCCT-3’
Fgfr3:	Fwd	5’-GGAGGACGTGGCTGAAGAC-3’

	Rev	5’-GGAGCTTGATGCCCCCAAT-3’
Fgfr4:	Fwd	5’-GCTCGGAGGTAGAGGTCTTGT-3’
	Rev	5’-CCACGCTGACTGGTAGGAA-3’
Gdf10:	Fwd	5’-CAGGACATGGTCGCTATCCAC-3’
	Rev	5’-ACAGGCTTTTGGTCGATCATTTC-3’
Hgf:	Fwd	5’-ATGTGGGGGACCAAACTTCTG-3’
	Rev	5’-GGATGGCGACATGAAGCAG-3’
Hprt:	Fwd	5’-AACAAAGTCTGGCCTGTATCCAA-3’
	Rev	5’-GCAGTACAGCCCCAAAATGG-3’
Jun:	Fwd	5’-CCTTCTACGACGATGCCCTC-3’
	Rev	5’-GGTTCAAGGTCATGCTCTGTTT-3’
Krt19:	Fwd	5’-GGGGGTTCAGTACGCATTGG-3’
	Rev	5’-GAGGACGAGGTCACGAAGC-3’
Lrat:	Fwd	5’-GAACCGTCCCTATGAAATCAGC-3’
	Rev	5’-GTCAGGCATTAGATGGGCGAC-3’
Myc:	Fwd	5’-TCTCCATCCTATGTTGCGGTC-3’
	Rev	5’-TCCAAGTAACTCGGTCATCATCT-3’
Ngfr:	Fwd	5’- CTAGGGGTGTCCTTTGGAGGT-3’
	Rev	5’- CAGGGTTCACACACGGTCT-3’
Tgfb1:	Fwd	5’-TTGCTTCAGCTCCACAGAGA-3’
	Rev	5’-TGGTTGTAGAGGGCAAGGAC-3’
Tgfb2:	Fwd	5’-CTTCGACGTGACAGACGCT-3’
	Rev	5’-GCAGGGGCAGTGTAAACTTATT-3’
Tgfb3:	Fwd	5’-CAGGCCAGGGCAGTCAGAG-3’

	Rev	5’-ATTTCCAGCCTAGATCCTGCC-3’

Human primers:

ACTA2:	Fwd:	5’-GTGTTGCCCCTGAAGAGCAT-3’
	Rev:	5’-GCTGGGACATTGAAAGTCTCA-3’
COL1A1:	Fwd:	5’-GAGGGCCAAGACGAAGACATC-3’
	Rev	5’-CAGATCACGTCATCGCACAAC-3’
FGF18:	Fwd:	5’-AGCCACATCGCTCAGACAC-3’
	Rev	5’-GCCCAATACGACCAAATCC-3’
GAPDH:	Fwd:	5’-TGCTTCCAGGTACAGGTGCT-3’
	Rev	5’-GCTGCTTACGGCTCACATCG-3’

Supplementary References

1. Tsuchida T*, et al.* A simple diet- and chemical-induced murine NASH model with rapid progression of steatohepatitis, fibrosis and liver cancer. *J Hepatol* **69**, 385-395 (2018).

2. Yang W*, et al.* Single-Cell Transcriptomic Analysis Reveals a Hepatic Stellate Cell-Activation Roadmap and Myofibroblast Origin During Liver Fibrosis in Mice. *Hepatology* **74**, 2774-2790 (2021).
