# Supplementary material for: Fibroblast growth factor 18 stimulates the proliferation of hepatic stellate cells, thereby inducing liver fibrosis
Source: Nat Commun. 2023 Oct 9;14:6304. doi: 10.1038/s41467-023-42058-z (PMC10562492; doi:10.1038/s41467-023-42058-z)
Supplement: Supplementary file 3 — Description of Additional Supplementary Files [file 41467_2023_42058_MOESM3_ESM.pdf]

Description of Additional Supplementary Files

Supplementary Data 1. Genes upregulated more than 2-fold in the livers of CflarLKO mice compared to CflarFF mice fed the CDE diet for 4 weeks.

Supplementary Data 2. Genes upregulated more than 2-fold in the livers of Fgf18 Tg mice compared to non-Tg mice.

Supplementary Data 3. Elevated genes in the livers of both CflarLKO mice fed the CDE diet and Fgf18 Tg mice.

Supplementary Data 4. Signature genes in each cluster from pooled non-Tg and Fgf18 Tg mice.
