# Supplementary material for: Fibroblast growth factor 18 stimulates the proliferation of hepatic stellate cells, thereby inducing liver fibrosis
Source: Nat Commun. 2023 Oct 9;14:6304. doi: 10.1038/s41467-023-42058-z (PMC10562492; doi:10.1038/s41467-023-42058-z)
Supplement: Supplementary file 8 — Reporting Summary [file 41467_2023_42058_MOESM8_ESM.pdf]

nature portfolio

Corresponding author(s): Hiroyasu Nakano
Last updated by author(s): 2023-9-15

Reporting Summary

Nature Portfolio wishes to improve the reproducibility of the work that we publish. This form provides structure for consistency and transparency in reporting. For further information on Nature Portfolio policies, see our Editorial Policies and the Editorial Policy Checklist.

Statistics

For all statistical analyses, confirm that the following items are present in the figure legend, table legend, main text, or Methods section.

n/a	Confirmed	
☐	☒	The exact sample size (*n*) for each experimental group/condition, given as a discrete number and unit of measurement
☐	☒	A statement on whether measurements were taken from distinct samples or whether the same sample was measured repeatedly
☐	☒	The statistical test(s) used AND whether they are one- or two-sided<br>*Only common tests should be described solely by name; describe more complex techniques in the Methods section.*
☒	☐	A description of all covariates tested
☐	☒	A description of any assumptions or corrections, such as tests of normality and adjustment for multiple comparisons
☐	☒	A full description of the statistical parameters including central tendency (e.g. means) or other basic estimates (e.g. regression coefficient) AND variation (e.g. standard deviation) or associated estimates of uncertainty (e.g. confidence intervals)
☐	☒	For null hypothesis testing, the test statistic (e.g. *F*, *t*, *r*) with confidence intervals, effect sizes, degrees of freedom and *P* value noted<br>*Give P values as exact values whenever suitable.*
☒	☐	For Bayesian analysis, information on the choice of priors and Markov chain Monte Carlo settings
☒	☐	For hierarchical and complex designs, identification of the appropriate level for tests and full reporting of outcomes
☒	☐	Estimates of effect sizes (e.g. Cohen's *d*, Pearson's *r*), indicating how they were calculated

Our web collection on statistics for biologists contains articles on many of the points above.

Software and code

Policy information about availability of computer code

Data collection	1)All-in-one microscope BZ-X700 and BZ-X Viewer v1.0.0 (Keyence), and confocal laser microscope LSM880 and ZEN software 2.3 SP1 FP_3 (Zeiss) - used to obtain images of mouse tissues<br>2)Amersham Biosciences imager 600 (Cytiva) - used for Western blotting analysis<br>3)7500 Real-time PCR detection system and 7500 SDS software v2.3 (Applied Biosystems) - used for quantitative PCR analysis<br>4)NovaSeq 6000 (Illumina) - used for sc-RNA-seq and RNA-seq analysis<br>5)SPECTRAmax 340PC and SoftMaxPro v4.8 (MolecularDevices) - used for colorimetric assays<br>6)BD RhapsodyTM Single-Cell Analysis System (BD Biosciences) - used for single cell isolation<br>7)HPLC; 1260 infinity series (Agilent), Column; Ascentis Express C18 (Supelco), and MS; 6120 Quadruple (Agilent) - used for bile acid analysis
Data analysis	1)7500 SDS software v2.3 (Applied Biosystems) - used for quantitative PCR analysis<br>2)FlowJo v10 (FlowJo) - used for flow cytmetric data analysis<br>3)Prism v9 (GraphPad software) - used to generate graphs and perform statistic analysis<br>4)BZ-X Analyzer 1.4.0.1 (Keyence), ZEN software 2.3 SP1 FP_3 (Zeiss), and Adobe Photoshop 24.7.0 (Adobe) - used to analyze images of mouse tissues<br>5)R3.6.1 with Genefilter and Gplots libraries - used to generate heatmaps<br>6)TCC-GUI v2021.11.13 (https://github.com/swsoyee/TCC-GUI) - used to normalize sequence count data and conduct differential gene-expression analysis<br>7)DAVID v6.8 (https://david.ncifcrf.gov/summary.jsp) - used for the functional annotations of differentially expressed genes<br>8)Seurat v4.1.1 (https://satijalab.org/seurat/), R v4.1.3 and CellChat (https://github.com/sqjin/CellChat) - used for the analysis of single cell RNA-seq data

9)Bowtie2 v2.4.2 (Johns Hopkins University) - used for mapping RNA-seq data to mouse reference genome
10)SoftMaxPro v4.8 (MolecularDevices) - used for colorimetric assays
11)TAS-Seq v1.0.0 (https://github.com/s-shichino1989/TASSeq) - a pipeline used for single cell RNA-seq analysis (Shichino et al., TAS-Seq is a robust and sensitive amplification method for bead-based scRNA-seq, Commun. Biol. 5, 602, 2022).

For manuscripts utilizing custom algorithms or software that are central to the research but not yet described in published literature, software must be made available to editors and reviewers. We strongly encourage code deposition in a community repository (e.g. GitHub). See the Nature Portfolio guidelines for submitting code & software for further information.

Data

Policy information about availability of data

All manuscripts must include a data availability statement. This statement should provide the following information, where applicable:

- Accession codes, unique identifiers, or web links for publicly available datasets
- A description of any restrictions on data availability
- For clinical datasets or third party data, please ensure that the statement adheres to our policy

Our bulk RNA-seq data of CflarFF, CflarLKO, NonTg, and Fgf18Tg mice were deposited in the GEO repository under the accession code GSE188273.
Our single-cell RNA seq data for analysis are available from the GEO repository under the accession number GSE205871.
The mouse reference genome GRCm39 and reference transcriptome GRCm38-101 were used for bulk RNA-seq and single cell RNA-seq, respectively.
The authors declare that the data supporting the findings of this study are available within the paper and its supplementary information files. Data not included are available from the corresponding authors upon reasonable request.
Source data is provided with this paper as Excel file and includes all the figures and Extended Data figures.

Research involving human participants, their data, or biological material

Policy information about studies with human participants or human data. See also policy information about sex, gender (identity/presentation), and sexual orientation and race, ethnicity and racism.

Reporting on sex and gender	The sex of human participants (8 male, 15 female) were based on self-reporting. We did not collect the gender information of the patients.
Reporting on race, ethnicity, or other socially relevant groupings	We did not collect the information of race, ethnicity, or other socially relevant groupings of the patients.
Population characteristics	Human participants were patients admitted to Juntendo University hospital to diagnose liver diseases. Age and genotypic information were not considered in this study.
Recruitment	Human participants were patients admitted to Juntendo University hospital to diagnose liver diseases. Informed consent was obtained from the patients before liver biopsy. Self-selection bias or other biases may not be present in this study.
Ethics oversight	Human study was approved by the ethics committee of Faculty of Medicine and Graduate School of Medicine, Juntendo University (E22-0085-H01).

Note that full information on the approval of the study protocol must also be provided in the manuscript.

Field-specific reporting

Please select the one below that is the best fit for your research. If you are not sure, read the appropriate sections before making your selection.

[x] Life sciences [ ] Behavioural & social sciences [ ] Ecological, evolutionary & environmental sciences

For a reference copy of the document with all sections, see nature.com/documents/nr-reporting-summary-flat.pdf

Life sciences study design

All studies must disclose on these points even when the disclosure is negative.

Sample size	We did not performed sample size calculation. Sample size was determined to be adequate based on the magnitude and consistency of measurable differences between groups. Sample size for each experiment was described in the manuscript. At least two independent experiments were performed to draw conclusion.
Data exclusions	No data was excluded in all of our experiments.
Replication	We confirmed that all attempts at replication were successful. At least two independent experiments were performed to draw conclusions.
Randomization	In the experiments of genetically modified mice, genotypes were checked at 3 weeks after birth, and the mice of appropriate genotypes were selected and used for analysis. Unhealthy mice were excluded from the study, and healthy mice were randomly assigned to control and experimental groups. Wild type mice were purchased from the breeder and randomly assigned to control and experimental groups. For in vitro experiments, control and experimental groups were always randomly assigned.

Blinding	Investigators were not blinded. However, data analysis was strictly quantitative, and no subjective decision making was necessary for all the experiments. Therefore, blinding in this study.

Reporting for specific materials, systems and methods

We require information from authors about some types of materials, experimental systems and methods used in many studies. Here, indicate whether each material, system or method listed is relevant to your study. If you are not sure if a list item applies to your research, read the appropriate section before selecting a response.

Materials & experimental systems

n/a	Involved in the study
☐	☒ Antibodies
☐	☒ Eukaryotic cell lines
☒	☐ Palaeontology and archaeology
☐	☒ Animals and other organisms
☒	☐ Clinical data
☒	☐ Dual use research of concern
☒	☐ Plants

Methods

n/a	Involved in the study
☒	☐ ChIP-seq
☐	☒ Flow cytometry
☒	☐ MRI-based neuroimaging

Antibodies

Antibodies used

Immunohistochemistry
anti-cleaved caspase-3 (9664, Cell Signaling, 1:1000)
anti-Ki67 (ab16667, Abcam, 1:200)
anti-CK19 (In-house, 1:200)
anti-CK19 clone TROMA-3 (MABT913, Merck, 1:1000)
anti-CD34 (Ab81289, Abcam, 1:1000)
anti-desmin (Ab32362, Abcam, 1:1,000)
anti-p75NTR (NGFR/TNFRSF16) (AF1157, R&D, 1:2000)
anti-CD68 (97778, Cell Signaling, 1:200)
anti-Ly-6G (87048, Cell Signaling, 1:200)
Biotinylated goat anti-rabbit IgG (E0432, DAKO, 1:200)
Alexa 594-conjugated anti-vimentin (677804, Biolegend, 1:400)
Alexa 647-conjugated donkey anti-rabbit IgG (A31573, Invitrogen, 1:500)
Alexa 488-conjugated donkey anti-rabbit IgG (A21206, Invitrogen, 1:500)
Alexa 488-conjugated donkey anti-goat IgG (A11055, Invitrogen, 1:500)

Western blotting
anti-FGF18 (120525, Wuhan Huamei Biotech, 1:1000)
anti-cFLIP (Dave-2, Adipogen, 1:1000)
anti-phospho-ERK (4370, Cell Signaling, 1:1000)
anti-ERK (4695, Cell Signaling, 1:1000)
anti-phospho-AKT (4060, Cell Signaling, 1:1000)
anti-AKT (4691, Cell Signaling, 1:1000)
anti-β-actin(SC-47778, Santa Cruz, 1:500))
HRP-conjugated donkey anti-rabbit IgG (NA934, GE Healthcare, 1:5000)
HRP-conjugated sheep anti-mouse IgG (NA931, ,GE Healthcare, 1:5000)

Flow cytometry
anti-mouse CD16/CD32 antibody (Bio X Cell, BE0307, 1:200)
BV421 anti-mouse CD45.2 (109832, Biolegend, 1:200)
APC anti-mouse CD34 (128611, Biolegend, 1:200)
APC anti-mouse Ly-6G (127614, Biolegend, 1:500)
FITC anti-mouse CD31 (102405, Biolegend, 1:200)
PE anti-mouse CD31 (102407, Biolegend, 1:100)
PE anti-mouse CD45.2 (109808, Biolegend, 1:100)
PE anti-mouse Thy1.2 (105307, Biolegend, 1:200)
PE anti-mouse Podoplanin (127407, Biolegend, 1:200)
FITC anti-mouse F4/80 (35-4801-U100, Tonbo Biosciences, 1:500)
FITC anti-mouse CD3ε (35-0031-U100, Tonbo Biosciences, 1:200)
PE anti-mouse B220 (50-0452-U100, Tonbo Biosciences, 1:200)
PE anti-mouse CD11b (50-0112-U100, Tonbo Biosciences, 1:500)
eBioscience™ Fixable Viability Dye eFluor™ 506 (65-0866-14, eBioscience, 1:5000)
PE anti-mouse PDGFRα (562776, BD biosciences, 1:200)

PE anti-mouse Sca1 (561076, BD biosciences, 1:200)

scRNA-seq
APC anti-mouse CD31 (17-0311-82, Invitrogen, 1:100)
APC anti-mouse CD45.2 (558702, BD biosciences, 1:100)
APC anti-mouse CD146 (134712, Biolegend, 1:100)
APC anti-mouse EpCAM (563478, BD biosciences, 1:100)
APC anti-mouse Ter119 (20-5921-U100, Tonbo Biosciences, 1:100)
anti-APC micro-Beads (130-090-855, Miltenyi, 1:5)
anti-PE micro-Beads UltraPure (130-105-639, Miltenyi, 1:5)
anti-MHC class I sampletag 1 (BD biosciences, 1:50)
anti-MHC class I sampletag 2 (BD biosciences, 1:50)
anti-MHC class I sampletag 3 (BD biosciences, 1:50)
anti-MHC class I sampletag 4 (BD biosciences, 1:50)
anti-MHC class I sampletag 5 (BD biosciences, 1:50)
anti-MHC class I sampletag 6 (BD biosciences, 1:50)

ELISA
Rat monoclonal anti-human FGF18 (12G7-9, in-house)
Rabbit monoclonal anti-human FGF18 (19S-SE5, in-house)

Validation

All primary antibodies from commercial vendors are validated by the manufacturers for the species and assay in our study. Validation data are available from the manufacturers' websites as follows:

Immunohistochemistry
anti-cleaved caspase-3 (9664, Cell Signaling, 1:1000)
Species:Human, Mouse, Rat, Monkey
Application: WB (1:1,000), IP (1:50), IHC-P (1:2,000), ICC (1:400-1:1,600), flow cytometry (1:6,400)
https://www.cellsignal.jp/products/primary-antibodies/cleaved-caspase-3-asp175-5a1e-rabbit-mab/9664

anti-Ki67 (ab16667, Abcam, 1:200)
Species: Human, Mouse, Rat
Application: flow cytometry (1:1,000), IHC-P (1:200), WB, ICC (1:250)
https://www.abcam.co.jp/products/primary-antibodies/ki67-antibody-sp6-ab16667.html

anti-CK19 clone TROMA-3 (MABT913, Merck, 1:1000)
Species: Human, Mouse
Application: WB, EM, IHC-P, IP, IF
https://www.merckmillipore.com/JP/ja/product/Anti-Cytokeratin-19-Antibody-clone-TROMA-3,MM_NF-MABT913-25UGG

anti-CD34 (Ab81289, Abcam, 1:1000)
Species: Human, Mouse, Rat
Application: WB (1:10,000), IHC-P (1:2,500-1:5,000), ICC (1:100-1:250), IP (1:30), flow cytometry (1:50)
https://www.abcam.co.jp/products/primary-antibodies/cd34-antibody-ep373y-ab81289.html

anti-desmin (Ab32362, Abcam, 1:1,000)
Species: Human, Mouse, Rat, Guinea Pig
Application: WB (1:100,000), IHC-P (1:2,000), flow cytometry (1:70), ICC (1:100-1:1,000),
https://www.abcam.co.jp/products/primary-antibodies/desmin-antibody-y66-cytoskeleton-marker-ab32362.html

anti-p75NTR (NGFR/TNFRSF16) (AF1157, R&D, 1:2000)
Species: Mouse
Application: WB (2 μg/mL), IHC (5-15 μg/mL)
https://www.rndsystems.com/products/mouse-ngfr-tnfrsf16-antibody_af1157

anti-CD68 (97778, Cell Signaling, 1:200)
Species: Mouse
Application: WB (1:1,000), IHC-P (1:150-1:600), ICC (1:400-1:1,600), flow cytometry (1:50-1:200)
https://www.cellsignal.jp/products/primary-antibodies/cd68-e3o7v-rabbit-mab/97778

anti-Ly-6G (87048, Cell Signaling, 1:200)
Species: Mouse
Application: WB (1:1,000), IHC (1:75-1:300)
https://www.cellsignal.jp/products/primary-antibodies/ly-6g-e6z1t-rabbit-mab/87048

Alexa 594-conjugated anti-vimentin (677804, Biolegend, 1:400)
Species: Human
Application: ICC:quality tested, IHC-P:verified
https://www.biolegend.com/ja-jp/products/alexa-fluor-594-anti-vimentin-antibody-12146?GroupID=GROUP26

Western blotting
anti-FGF18 (120525, Wuhan Huamei Biotech, 1:1000)
Species: Human, Mouse, Rat
Application: IHC (1:25-1:100), ELISA (1:1,000-1:2,000)
https://www.cusabio.com/Polyclonal-Antibody/Rabbit-anti-human-FGF18-polyclonal-antibody-11101386.html
Note: FGF18 is a secreted protein, and after our extensive experimental verification, we concluded that IHC of FGF18 in tissues is extremely difficult. In fact, this antibody nonspecifically stained hepatocytes in hepatocyte-specific Fgf18-deficient mouse liver sections used in our experiments. However, we verified that this antibody can be used for WB and ELISA of human and mouse FGF18 (Tsuchiya et al., A high-sensitivity ELISA for detection of human FGF18 in culture supernatants from tumor cell lines. Biochem. BIophys. Res. Commun. 675:71-77, 2023).

anti-cFLIP (Dave-2, Adipogen, 1:1000)
Species: Human, Mouse
Application: WB, IP
https://adipogen.com/ag-20b-0005-anti-flip-mab-dave-2.html/

anti-phospho-ERK (4370, Cell Signaling, 1:1000)
Species: Human, Mouse, Rat, Hamster, Monkey, Mink, D.melanogaster, Zebrafish, Bovine, Dog, Pig, S.cerevisiae
Application: WB (1:2,000), IP (1:50), IHC-P (1:200-1:800), ICC (1:200-1:400), flow cytometry (1:800-1:1,600)
https://www.cellsignal.jp/products/primary-antibodies/phospho-p44-42-mapk-erk1-2-thr202-tyr204-d13-14-4e-xp-rabbit-mab/4370

anti-ERK (4695, Cell Signaling, 1:1000)
Species: Human, Mouse, Rat, Hamster, Monkey, Mink, D.melanogaster, Zebrafish, Bovine, Dog, Pig, C.elelgans
Application: WB (1:1,000), IP (1:50), IHC-P (1:125-1:250), ICC (1:400-1:1,600), flow cytometry (1:200-1:800)
https://www.cellsignal.jp/products/primary-antibodies/p44-42-mapk-erk1-2-137f5-rabbit-mab/4695

anti-phospho-AKT (4060, Cell Signaling, 1:1000)
Species: Human, Mouse, Rat, Hamster, Monkey, D.melanogaster, Zebrafish, Bovine
Application: WB (1:2,000), IP (1:50), IHC-P (1:50-1:200), ICC (1:400-1:800), flow cytometry (1:100-1:400)
https://www.cellsignal.jp/products/primary-antibodies/phospho-akt-ser473-d9e-xp-rabbit-mab/4060

anti-AKT (4691, Cell Signaling, 1:1000)
Species: Human, Mouse, Rat, Monkey, D.melanogaster
Application: WB (1:1,000), IP (1:50), IHC-P (1:150-1:600), ICC (1:200-1:800), flow cytometry (1:100-1:400)
https://www.cellsignal.jp/products/primary-antibodies/akt-pan-c67e7-rabbit-mab/4691

anti-β-actin(SC-47778, Santa Cruz, 1:500))
Species: Human, Mouse, Rat, Avian, Bovine, Canine, Porcine, Rabbit, D.discoideum, P.polycephalum
Application: WB, IP, IF, IHC-P, ELISA
https://www.scbt.com/ja/p/beta-actin-antibody-c4

Flow cytometry
anti-mouse CD16/CD32 antibody (Bio X Cell, BE0307, 1:200)
Species: Mouse
Application: Fc receptor blocking
https://bioxcell.com/invivomab-anti-mouse-cd16-cd32

BV421 anti-mouse CD45.2 (109832, Biolegend, 1:200)
Species: Mouse
Application: flow cytometry
https://www.biolegend.com/ja-jp/products/brilliant-violet-421-anti-mouse-cd45-2-antibody-7328?GroupID=BLG7007

APC anti-mouse CD34 (128611, Biolegend, 1:200)
Species: Mouse
Application: flow cytometry
https://www.biolegend.com/ja-jp/products/apc-anti-mouse-cd34-antibody-6520

APC anti-mouse Ly-6G (127614, Biolegend, 1:500)
Species: Mouse
Application: flow cytometry
https://www.biolegend.com/ja-jp/products/apc-anti-mouse-ly-6g-antibody-6115

FITC anti-mouse CD31 (102405, Biolegend, 1:200)
Species: Mouse
Application: flow cytometry
https://www.biolegend.com/ja-jp/products/fitc-anti-mouse-cd31-antibody-120

PE anti-mouse CD31 (102407, Biolegend, 1:100)

Species: Mouse
Application: flow cytometry
https://www.biolegend.com/ja-jp/products/pe-anti-mouse-cd31-antibody-122

PE anti-mouse CD45.2 (109808, Biolegend, 1:100)
Species: Mouse
Application: flow cytometry
https://www.biolegend.com/ja-jp/products/pe-anti-mouse-cd45-2-antibody-7

PE anti-mouse Thy1.2 (105307, Biolegend, 1:200)
Species: Mouse
Application: flow cytometry
https://www.biolegend.com/ja-jp/products/pe-anti-mouse-cd90-2-thy1-2-antibody-106

PE anti-mouse Podoplanin (127407, Biolegend, 1:200)
Species: Mouse
Application: flow cytometry
https://www.biolegend.com/ja-jp/products/pe-anti-mouse-podoplanin-antibody-4882

FITC anti-mouse F4/80 (35-4801-U100, Tonbo Biosciences, 1:500)
Species: Mouse
Application: flow cytometry
https://cytekbio.com/products/fitc-anti-mouse-f4-80-antigen-bm8-1?variant=40581222858788

FITC anti-mouse CD3ε (35-0031-U100, Tonbo Biosciences, 1:200)
Species: Mouse
Application: flow cytometry
https://cytekbio.com/products/fitc-anti-mouse-cd3e-145-2c11?variant=40581224136740

PE anti-mouse B220 (50-0452-U100, Tonbo Biosciences, 1:200)
Species: Mouse
Application: flow cytometry
https://cytekbio.com/products/pe-anti-human-mouse-cd45r-b220-ra3-6b2?variant=40581210275876

PE anti-mouse CD11b (50-0112-U100, Tonbo Biosciences, 1:500)
Species: Human, Mouse
Application: flow cytometry
https://cytekbio.com/products/pe-anti-human-mouse-cd11b-m1-70?variant=40581210374180

PE anti-mouse PDGFRα (562776, BD biosciences, 1:200)
Species: Mouse
Application: flow cytometry
https://www.bdbiosciences.com/ja-jp/products/reagents/flow-cytometry-reagents/research-reagents/single-color-antibodies-ruo/pe-rat-anti-mouse-cd140a.562776

PE anti-mouse Sca1 (561076, BD biosciences, 1:200)
Species: Mouse
Application: flow cytometry
https://www.bdbiosciences.com/ja-jp/products/reagents/flow-cytometry-reagents/research-reagents/single-color-antibodies-ruo/pe-rat-anti-mouse-ly-6a-e.561076

scRNA-seq
APC anti-mouse CD31 (17-0311-82, Invitrogen, 1:100)
Species: Mouse
Application: flow cytometry
https://www.thermofisher.com/antibody/product/CD31-PECAM-1-Antibody-clone-390-Monoclonal/17-0311-82

APC anti-mouse CD45.2 (558702, BD biosciences, 1:100)
Species: Mouse
Application: flow cytometry
https://www.bdbiosciences.com/ja-jp/products/reagents/flow-cytometry-reagents/research-reagents/single-color-antibodies-ruo/apc-mouse-anti-mouse-cd45-2.558702

APC anti-mouse CD146 (134712, Biolegend, 1:100)
Species: Mouse
Application: flow cytometry
https://www.biolegend.com/de-de/products/apc-anti-mouse-cd146-antibody-9289?GroupID=BLG10622

APC anti-mouse EpCAM (563478, BD biosciences, 1:100)

Species: Mouse
Application: flow cytometry
https://www.bdbiosciences.com/ja-jp/products/reagents/flow-cytometry-reagents/research-reagents/single-color-antibodies-ruo/apc-rat-anti-mouse-cd326.563478

APC anti-mouse Ter119 (20-5921-U100, Tonbo Biosciences, 1:100)
Species: Mouse
Application: flow cytometry
https://cytekbio.com/products/apc-anti-mouse-ter-119-ter-119?variant=40581235376164

anti-MHC class I sampletag 1 (BD biosciences, 1:50)
anti-MHC class I sampletag 2 (BD biosciences, 1:50)
anti-MHC class I sampletag 3 (BD biosciences, 1:50)
anti-MHC class I sampletag 4 (BD biosciences, 1:50)
anti-MHC class I sampletag 5 (BD biosciences, 1:50)
anti-MHC class I sampletag 6 (BD biosciences, 1:50)
Species: Mouse
Application: Direct cell labeling for scRNA-seq
https://www.bdbiosciences.com/ja-jp/products/reagents/single-cell-multiomics/single-cell-multiplexing-kit

anti-CK19 (In-house, 1:200)
Rabbit polyclonal anti-mouse CK19 in-house antibody was raised against the C-terminal peptide of mouse CK19 and was validated for IHC-P in the previous publication (Tanimizu et al., Isolation of hepatoblasts based on the expression of Dlk/Pref-1. J. Cell Sci. 116, 1775-1786, 2003). We also verified that this antibody can be used for WB and ICC.

Rat monoclonal anti-human FGF18 (12G7-9, in-house)
Rabbit monoclonal anti-human FGF18 (19S-SE5, in-house)
Rat and rabbit monoclonal anti-human FGF18 antibodies were validated by us using commercial recombinant human FGF18 (PeproTech 100-28) as a standard (Tsuchiya et al., A high-sensitivity ELISA for detection of human FGF18 in culture supernatants from tumor cell lines. Biochem. BIophys. Res. Commun. 675:71-77, 2023). These antibodies detected mouse FGF18 only weakly.

Eukaryotic cell lines

Policy information about cell lines and Sex and Gender in Research

Cell line source(s)	KTPU8 is a feeder-free ES cell line derived from TT2 ES cell line. TT2 ES cell line is widely used in Japan which was established from F1 embryo of C57BL/6 and CBA.
Authentication	KTPU8 cell line was established by Nakahara et al. and authenticated (Nakahara et al., Gene-trap mutagenesis using Mol/MSM-1 embryonic stem cells from MSM/Ms mice. Mamm Genome 24:228-239, 2013).
Mycoplasma contamination	The cell line was not tested for mycoplasma contamination.
Commonly misidentified lines (See ICLAC register)	Commonly misidentified lines were not used in this study.

Animals and other research organisms

Policy information about studies involving animals; ARRIVE guidelines recommended for reporting animal research, and Sex and Gender in Research

Laboratory animals	All mouse strains used in this study are C57BL/6 origin and described in the Material and Methods sections. The mice were housed in 23±2℃, a humidity of 55%±5%, and a 12 hours dark/light cycle. Feeding Experiments were started at 8 weeks for 4 weeks (CDE and DDC) or 12 weeks (CDAHFD). Mice fed normal diet were analyzed at 12 weeks. Female mice were used for CDE treatment, whereas male mice were used for DDC treatment. Both male and female mice were used for other experiments. Non-Tg and Fgf18Tg mice were fed normal diet and analyzed at young (6-8 weeks) and adult (18-24 weeks) ages.
Wild animals	This study did not use wild animals.
Reporting on sex	Only female mice were used for CDE diet experiments, because our preliminary analysis indicated that CDE diet-induced hepatitis were more severe in female mice than in male mice. Only male mice were used for DDC diet experiments, because male mice generally give more reproducible results due to the lack of estrous cycle. We did not test whether female mice give similar results. In all other experiments (normal diet, CDAHFD, non-Tg and Fgf18Tg), both male and female mice were used and gave similar results.
Field-collected samples	This study did not use field-collected samples.
Ethics oversight	All experiments were performed according to the guidelines approved by the Institutional Animal experiments Committee of Juntendo University School of Medicine (250071) and Toho University Faculty of Medicine (21-53-409 and 21-53-412).

Note that full information on the approval of the study protocol must also be provided in the manuscript.

Flow Cytometry

Plots

Confirm that:

- [x] The axis labels state the marker and fluorochrome used (e.g. CD4-FITC).
- [x] The axis scales are clearly visible. Include numbers along axes only for bottom left plot of group (a 'group' is an analysis of identical markers).
- [x] All plots are contour plots with outliers or pseudocolor plots.
- [x] A numerical value for number of cells or percentage (with statistics) is provided.

Methodology

Sample preparation

To purify liver non-parenchymal cells, we performed a modified two-step collagenase perfusion method as described previously. Briefly, the livers from 6- to 8-week-old non-Tg and Fgf18 Tg mice were perfused with the liver perfusion medium (17701-038, ThermoFisher Scientific) at a flow rate of 3 ml/min for 5 min. Then, the livers were perfused with the basic perfusion solution (136 mM NaCl, 5.4 mM KCl, 5 mM CaCl2, 0.5 mM NaH2PO3 2H2O, 0.42 mM Na2HPO3, 10 mM HEPES pH 7.5, 5 mM glucose and 4.2 mM NaHCO3) containing 0.5 mg/ml collagenase type IV (C5138, Sima-Aldrich) and 0.06 mg/ml DNase I (DN25, Sigma-Aldrich) at a flow rate of 3 ml/min for 8 min. The digested livers were transferred to a glass dish, and fibrous connective tissues were removed from the livers with a pair of tweezers. For Hep-Fgf18 Tg mice, perfusion of livers with the basic perfusion solution alone was not sufficient to isolate liver non-parenchymal cells from fibrotic livers. Thus, after perfusion, we further incubated the livers with the basic perfusion solution using a stirrer bar at 37℃ for 5 min. Cells were dispersed by pipetting and were passed through a 70 µm cell strainer. After centrifugation at 500 rpm for 1 min, the supernatant was transferred to a new tube. The pellet was resuspended with DMEM and centrifuged at 500 rpm for 1 min; then, the pellet was used as hepatocytes. The new supernatant was combined with the former supernatant and then centrifuged at 700 rpm for 2 min several times until no cell pellet was visible. The final supernatant was centrifuged at 1800 rpm for 5 min, and the pellets were resuspended in RBC-lysis solution (0.17 mM NH4Cl, 0.01 mM EDTA, 0.1 M Tris, pH 7.3) and then washed twice in DMEM medium containing 10% FCS. Cells were then subjected to flow cytometry or cell sorting.
In single cell isolation for scRNA-seq, we isolated non-parenchymal cells from the livers of 6-week-old non-Tg and Fgf18 Tg mice. To enrich HSCs and fibroblasts, we depleted lineage marker-positive cells using APC-conjugated antibodies that react with the following lineage markers (CD31, CD45.2, CD146, EpCAM, and Ter119), followed by anti-APC micro-beads and LS columns (130-042-401, Miltenyi). Following depletion, approximately 5 x 104 cells were stained with different DNA barcode-conjugated MHC class 1 antibodies (BDTM Mouse Immune Single-Cell Multiplexing Kit [MHC H2 Class I], 626545, BD Biosciences) to identify each mouse. Then, lineage-negative cells were sorted by BD FACSAriaTM III (BD Biosciences).

Instrument

LSRFortessa X-20 (BD biosciences)
FACSAria Fusion (BD biosciences)

Software

BD FACSDiva Software Version 8.0.1 (BD biosciences)
FlowJo software Version 10.6 (FlowJo)

Cell population abundance

In hepatic non-parenchymal cell analysis, at least 70,000 live cells were analyzed.
In cell sorting, at least 200,000 live cells were obtained.
In single cell isolation for scRNA-seq, 4,000 live cells from 3 Non-Tg and 3 Fgf18Tg mice were separately obtained, barcode-labeled, and mixed (total 24,000 cells).

Gating strategy

For hepatic non-parenchymal cell analysis (Fig.5e-g and Supplementary Fig. 7g-7i),
1) Cells were identified based on FSC-A/SSC-A.
2) Doublets were excluded based on FSC-A/FSC-H.
3) Live cells were selected by gating on FVD506-A negative events.
4) Lymphocytes were selected by gating on CD45.2-BV421-A positive events.
5) Lymphocyte marker expression were analyzed in CD45.2-BV421-A positive events.
6) Endothelial cell and hepatic stellate cell marker expression were analyzed in CD45.2-BV421-A negative events.

For cell sorting (Fig. 5h and Supplementary Fig. 9a),
1) Cells were identified based on FSC-A/SSC-A.
2) Doublets were excluded based on FSC-A/FSC-H.
3) Live cells were selected by gating on FVD506-A negative events.
4) Endothelial cell and hepatic stellate cell were selected by gating on CD45.2-BV421-A negative events.

For single-cell isolation for single cell RNA-seq (Fig. 6 and Supplementary Fig. 10a),
1) Lineage markers (CD31, CD45.2, CD146, EpCAM, and Ter119)-positive cells were removed using APC-conjugated antibodies and magnetic beads.
2) Lineage-negative cells were identified based on FSC-A/SSC-A.
3) Doublets were excluded based on FSC-A/FSC-H.
4) Live cells were selected by gating on 7-AAD-negative events.
5) Lineage-negative cells were selected by gating on Lineage markers-APC negative events.

[x] Tick this box to confirm that a figure exemplifying the gating strategy is provided in the Supplementary Information.
